# A randomized controlled study of auricular point acupressure to manage chemotherapy-induced neuropathy: Study protocol

**DOI:** 10.1371/journal.pone.0311135

**Published:** 2024-09-26

**Authors:** Nada Lukkahatai, Michael V. Nguyen, Jingyu Zhang, Yu-Min Cho, Chitchanok Benjasirisan, Heijingzi Monica Jia, Claudia M. Campbell, Jennifer Kawi, Hulin Wu, Hongyu Wang, Rupsikha Bora, Johannes Thrul, Constance M. Johnson, Thomas J. Smith

**Affiliations:** 1 Johns Hopkins University School of Nursing, Baltimore, MD, United States of America; 2 Sidney Kimmel Comprehensive Cancer Center at Johns Hopkins, Baltimore, MD, United States of America; 3 The University of Texas Health Science Center at Houston, McGovern Medical School, Houston, TX, United States of America; 4 The University of Texas Health Science Center at Houston, Cizik School of Nursing, Houston, TX, United States of America; 5 Johns Hopkins Bloomberg School of Public Health, Baltimore, MD, United States of America; 6 Johns Hopkins University School of Medicine, Baltimore, MD, United States of America; 7 The University of Texas Health Science Center at Houston, School of Public Health, Houston, TX, United States of America; China Medical University, TAIWAN

## Abstract

**Objective:**

Chemotherapy-induced neuropathy (CIN) significantly impacts cancer patients, leading to functional disability, diminished quality of life, and increased healthcare costs amid the ongoing opioid crisis. Auricular point acupressure (APA), a non-invasive and non-pharmacological alternative, has shown potential for alleviating the pain, numbness, and tingling associated with CIN. This study aims to assess the efficacy of APA for CIN symptoms and physical function and to examine the mechanisms underlying APA’s effects on CIN.

**Methods:**

This is a three-arm randomized controlled clinical trial protocol. Patients aged 18 and older who are experiencing CIN are randomly assigned to one of the three groups: an APA group (in-person APA; mAPA), a sham control group (virtual APA; vAPA), and a wait-list usual care control group (UC). During the four-week program, participants in the mAPA receive an in-person APA treatment and training; the sham control participants (vAPA) receive a self-guided smartphone APA application with APA demonstration videos; and the UC participants will continue with the usual care and be re-randomized into one of the APA groups. The primary outcomes are changes in CIN symptoms and physical function. Secondary outcomes include evaluating pain sensory thresholds, motor and cognitive functioning, inflammatory signaling, brain connectivity, opioid use, and quality of life. The outcomes are measured at baseline, program completion (4 weeks), and at monthly follow-up for 3 months post-intervention.

**Discussion:**

This study will provide evidence supporting the potential viability of APA as an intervention for CIN.

**Trial registration:**

ClinicalTrials.gov, ID NCT04920097 registered on 3 June 2021.

## Introduction

More than 70% of cancer patients develop chemotherapy-induced neuropathy (CIN), a serious side effect associated with chemotherapy treatments, including platinum drugs, vinca alkaloids, taxanes, and bortezomib [1]. CIN is a significant sensory neuropathy characterized by pain, numbness, and tingling, which may also involve motor changes such as loss of strength. This complication can lead to delays or treatment discontinuation, or dose reductions, ultimately impacting survival rates [2]. With advancements in cancer treatments and increased survival, the long-term effects of CIN continue to impose a substantial burden, affecting up to 50% of cancer survivors six years post-treatment and increasing the risk of falls by 1.8 times [3]. CIN contributes to considerable functional disability, negatively affects the quality of life, and leads to high healthcare costs and resource utilization [2, 4].

Currently, there is no universally effective treatment for CIN. Pharmacotherapy, including opioids and gabapentin, is the predominant treatment approach. However, cancer patients are already subjected to multiple medications, leading to a heightened risk of various adverse effects. Ideally, patients could be provided with non-pharmacological options to manage CIN. Non-pharmacological interventions such as exercise, acupuncture, scramble therapy, electronic nerve stimulation, yoga, dietary supplements, and self-care strategies have been used with limited effects. Some interventions, such as acupuncture and scramble therapy, have shown promising CIN relief [5–7], but their use is limited because of high dropout rates due to frequent office visits, limited insurance coverage [8], and fear of needles [9].

Auricular point acupressure (APA) is derived from auricular acupuncture/auricular therapy. This theory was developed from Chinese medicine into modern science in the 1980s by Paul Nogier, MD [10, 11], by mapping a somatotopic representation of the human body onto the ear. Specific points of the ear correspond to specific organs and areas of the body. The symptomatic parts of the body can be treated by stimulating these ear points. In Dr. Nogier’s system of diagnosis, the location of the ear point corresponding to the symptomatic body is confirmed by electrodermal responses (i.e., an electrical point finder) [12, 13].

Once identified, these points can be stimulated—classically with needles, electrically [13, 14], or with APA [15]. The underlying theory of auricular acupuncture posits that nerves in the outer ear correspond to specific areas of the brain, and these areas have a reflex connection with specific parts of the body [13, 14]. Functional magnetic resonance imaging (fMRI) has validated the correlation between ear points and brain activity [16].

This study was built on evidence from our pilot data regarding APA [17–19]. We found that patients with CIN experienced reductions in pain severity (46%), numbness (49%), and tingling (38%) in the toes compared to baseline with the 4 weeks of APA intervention. Using Quantitative Sensory Testing (QST)—a well-recognized psychophysical tool to quantify pain and sensory nerve function [20]—our data showed that after 4 weeks of APA, participants reported higher toe sensitivity, indicating that toe numbness (a prime characteristic of CIN) had improved, and a higher pain threshold of suprathreshold pinprick stimuli on the index finger, suggesting that APA may enhance endogenous pain inhibition [17]. The fMRI data taken after 10 minutes of APA indicated an increased brain functional connectivity between the salience and basal ganglia networks (possibly explaining the immediate relief after APA administration) [18].

On delayed imaging 4 weeks post APA, we found increased connectivity in the executive control networks, which may directly or indirectly modulate the cognitive control of pain perception [21]. We also found significant changes in plasma inflammatory markers (IL-1β, IL-2, IL-6, TNFα, and IFNγ) after 4 weeks [19]. This finding suggests that APA may influence brain macrophage cells, leading to alterations in inflammatory cytokines [22], which could result in sustained CIN relief at the one-month follow-up. Although the exact pathophysiology of CIN remains unknown [23], evidence suggests the involvement of inflammation. Our data indicate that APA shows great promise to reverse CIN through neuronal modulation, as evidenced by functional connectivity changes (fMRI data), thereby blocking/releasing pro/anti-inflammatory cytokines into the peripheral nervous system to achieve CIN relief [17, 19].

**Fig 1:**
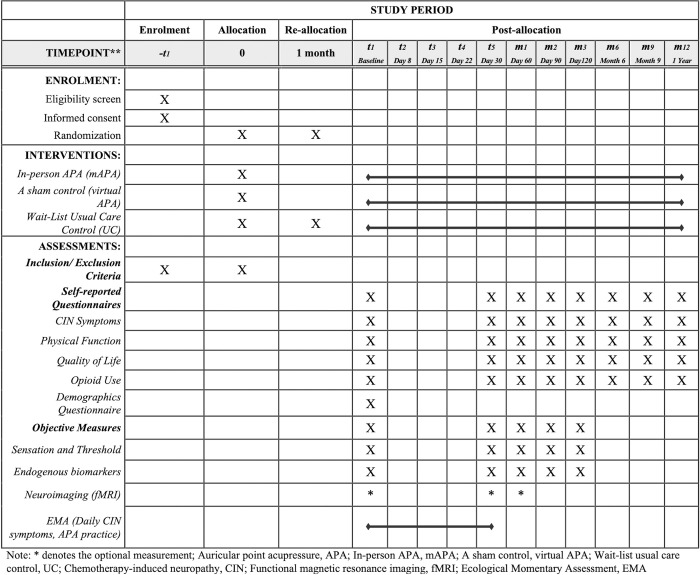
SPIRIT figure.

Building on these findings, we aim to refine the treatment protocol and conduct a study to further investigate and validate APA’s efficacy in managing CIN. Guided by the ’Standard Protocol Items: Recommendations for Interventional Trials’ (SPIRIT) guidelines (S1 Checklist), this manuscript describes our current treatment and a randomized controlled trial study protocol. Fig 1 provides a visual representation of the trial’s schedule and the participants’ time commitment.

### Theoretical framework

**Fig 2:**
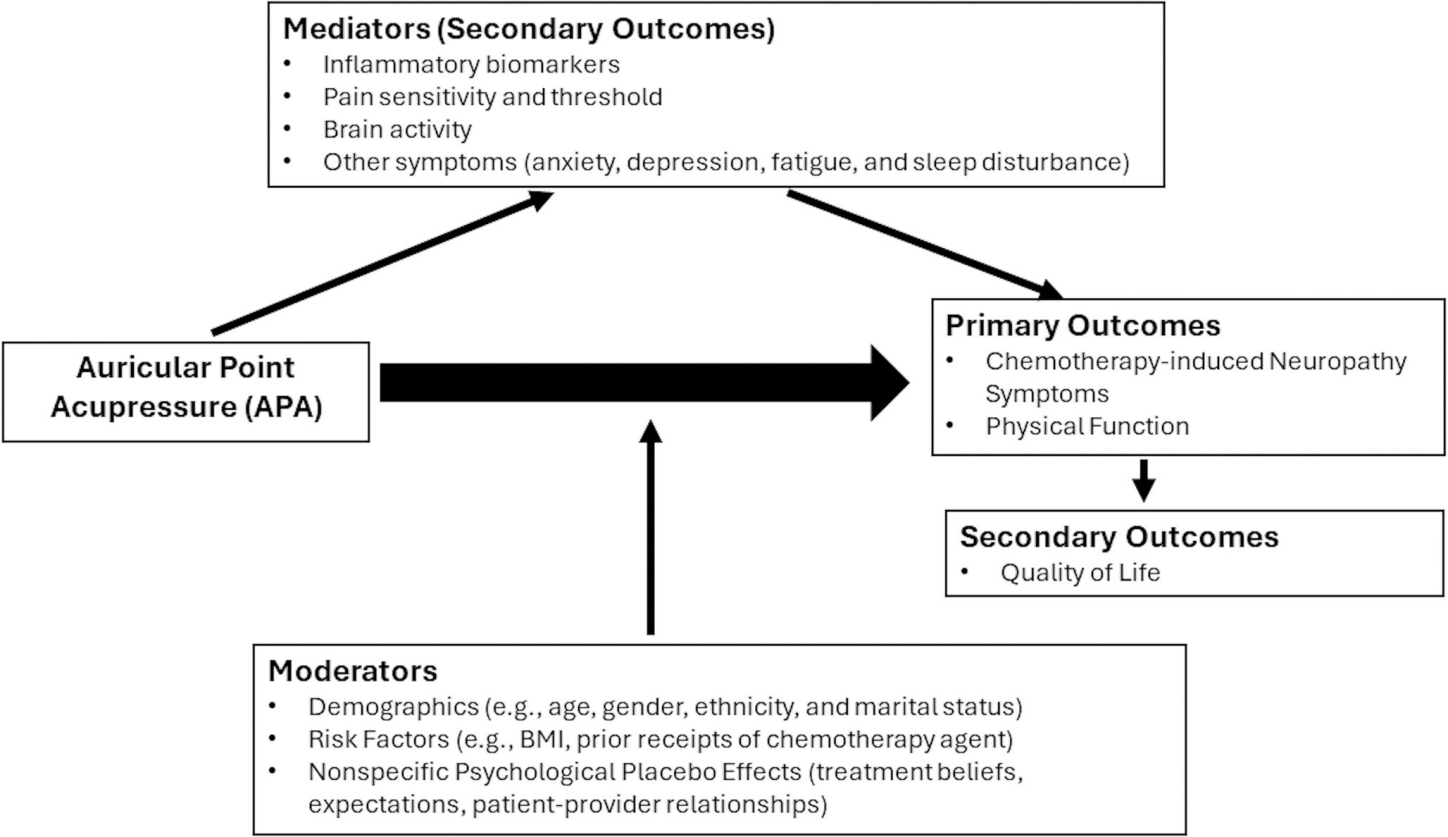
Effects of auricular point acupressure on chemotherapy-induced neuropathy framework. The framework, guided by the NIH Symptom Science Model, proposes that Auricular Point Acupressure affects Chemotherapy-Induced Neuropathy by modulating endogenous biomarkers, pain sensitivity, brain activity, and other cancer-related symptoms.

Guided by the NIH Symptom Science Model [24] and the biopsychosocial model of pain [25, 26], our framework (Fig 2) hypothesizes that the effects of APA on CIN are mediated by endogenous (inflammatory) biomarkers, pain sensitivity and threshold, and brain activity change, with additional contributions for other cancer-related symptoms [24, 27]. The evidence suggests that symptoms such as pain, anxiety, depression, fatigue, and sleep disturbance [28–30] (overall quality of life) are interrelated; therefore, we explore their contributions to APA effects on CIN outcomes.

Moreover, CIN effects are moderated by demographics [31, 32] and nonspecific psychological placebo effects, including beliefs in treatment efficacy [33, 34], treatment expectations [35, 36], and patient-provider relationships [37]. Additionally, because risk factors (BMI, prior receipt of chemotherapy agent) are associated with CIN [38], we examine their contribution to demographic factors (age, gender, ethnicity, and marital status) [39]. A comprehensive understanding of the role of these factors is crucial to developing more effective approaches to managing CIN.

This study aims to: 1) examine the efficacy of APA on CIN symptoms (e.g., pain, numbness, tingling, stiffness) and physical function (primary outcomes), and pain sensory and thresholds, motor and cognitive functioning, inflammatory signaling, brain connectivity, opioid use, and quality of life (secondary outcomes); 2) investigate the mechanisms underlying the beneficial/non-beneficial effects of APA on CIN symptoms, and 3) explore the sustained effect of APA for CIN on CIN symptoms and physical function at 1, 2, and 3 months post-APA.

## Materials and methods

### Design

This is a prospective randomized controlled study. Participants are randomly assigned into three groups: the in-person APA (mAPA) group, the sham control (virtual APA; vAPA) group, and the Wait-List Usual Care (UC) group. The mAPA group receives one in-person treatment and training session for the participant and/or caregiver to place the seeds, a self-guided smartphone application, and one secure Zoom session for APA coaching. The vAPA group uses a self-guided smartphone application and participates in one secure Zoom session for APA coaching, which includes questions and answers. The UC group serves as a wait-list control.

Participants attend one secure Zoom session for APA coaching one week after the training. Participants in the vAPA group are asked to review and follow instructional videos on seed placement and stimulation on the APA smartphone application. After the baseline visit, participants in the vAPA group received one Zoom session for APA coaching one week after the baseline study visit. Participants in the UC group continue with their usual care and are re-randomized into either the APA or sham control groups after the 4^th^-week visit (T2).

### Ethical approval and trial registration

The study received approval from The Johns Hopkins Medicine Institutional Review Boards (JHM IRBs) under the protocol IRB00243141. This study adheres to the principles outlined in the Declaration of Helsinki. Prior to participation, all participants provided written informed consent. The study has been reviewed and registered on ClinicalTrials.gov with the identifier NCT04920097.

### Recruitment

Participants are recruited at a comprehensive cancer center in Baltimore, Maryland, and a cancer center in Houston, Texas. The recruitment methods include (1) providers’ referrals, (2) flyers at cancer clinics, and letters and newsletters mailed or e-mailed to local oncologists, and (3) through collaborative relationships formed with oncologists. The consents are obtained by trained study coordinators. As of the current date, the study recruitment commenced on July 8th, 2021, and is ongoing.

### Participant eligibility

The study’s inclusion criteria include cancer patients aged 18 years or older who have undergone chemotherapy using platinum-based, vinca alkaloids, bortezomib, eribulin, immunomodulatory drugs (IMiDs) such as lenalidomide, and/or taxanes, and who have completed their chemotherapy regimen at least three months before enrollment.

Additionally, eligible participants must exhibit CIN or pre-existing peripheral neuropathy exacerbated by chemotherapy, experiencing an average intensity of pain, numbness, or tingling on their extremities rated ≥ 4 on an 11-point numerical scale in the previous week.

Exclusion criteria include individuals who have used investigational pain control agents concurrently or within the past 30 days, employed implantable drug delivery systems for pain management (e.g., Medtronic SynchroMed^®^), undergone prior neurolytic pain control treatments, such as celiac plexus blocks, suffered from other identifiable causes of painful paresthesia predating chemotherapy (e.g., radiation or malignant plexopathy, lumbar or cervical radiculopathy), or have a history of allergic reactions to latex or adhesive tape, rendering them ineligible for participation. The summary of inclusion and exclusion criteria is listed in Table 1.

**Table 1:** Summary of inclusion/exclusion criteria.

Criteria	Description
**Inclusion criteria**	
Age	Patients aged 18 years or older.
Medication Received	Received medication from one of the following categories: platinum-based, vinca alkaloids, bortezomib, eribulin, immunomodulatory drugs (IMiDs) such as lenalidomide, and/or taxanes
Time Since Chemotherapy Completion	Completed chemotherapy course at least three months before enrollment.
Neuropathy Etiology	Developed neuropathy due to neurotoxic chemotherapy for cancer or have pre-existing peripheral neuropathy exacerbated by chemotherapy.
Severity of Symptoms	Reported an average intensity of pain, numbness, or tingling in extremities rated ≥ 4 on an 11-point numerical scale during the previous week due to CIN.
**Exclusion criteria**	
Investigational Agent for Pain Control	Concurrent use of an investigational agent for pain control or use within the past 30 days.
Implantable Drug Delivery System	Managing pain using an implantable drug delivery system (e.g., Medtronic SynchroMed®)
Prior Neurolytic Pain Treatment	Previously undergone neurolytic pain control treatments, such as celiac plexus blocks.
Other potential causes painful paresthesia	Other identified causes of painful paresthesia present before chemotherapy, such as radiation or malignant plexopathy, lumbar or cervical radiculopathy.
Allergic Reactions to Latex and Adhesive Tape	History of allergic reactions (e.g., redness, swelling, itching, etc.) to latex and adhesive tape.

### Sample size and study power

The sample size was determined through power analysis to detect the intervention effects at one-month post-intervention, accounting for the drop-out rate. We used data from the preliminary study described above for our primary outcomes of lower extremity pain, numbness, and function.

Based on our pilot control group (waitlist), the effect sizes ranged from -0.42 and -0.85 [17]. With a statistical power of 0.80 and an alpha level of 0.05, considering these effect sizes, we need approximately 17 to 56 participants per group to detect significant differences between two groups (intervention group vs. Sham APA group or intervention group vs. Usual Care Control group) using a two-group repeated measures generalized linear model.

In our pilot studies, participants were successfully retained and did not drop out of the study before the second APA treatment [40, 41]. We had a 13% dropout (n = 2, total n = 15) at the 1-month follow-up in our CIN pilot study [17] and an 11% dropout of patients with breast cancer (n = 2, total n = 22) in our study of persistent post-mastectomy pain [42]. Around 25% of the enrolled participants were expected to be lost to follow-up [17]. Therefore, we enroll 225 participants (75 per group).

For neuroimaging, the effect size of 0.45 is within the effect size seen in our preliminary investigation of the effect of APA on fMRI at 4-week APA in our study with cancer patients with neuropathy [18]. In our preliminary investigation, the effect sizes associated with the basal ganglia and salience networks were -0.92 after a 4-week APA for fMRI and 0.66 for the sensation. For the fMRI component, we plan to recruit 36 participants (18 from the APA Group and 18 from Sham APA Control) to investigate brain activity changes due to APA.

### Study intervention

#### APA treatment protocol

**Fig 3:**
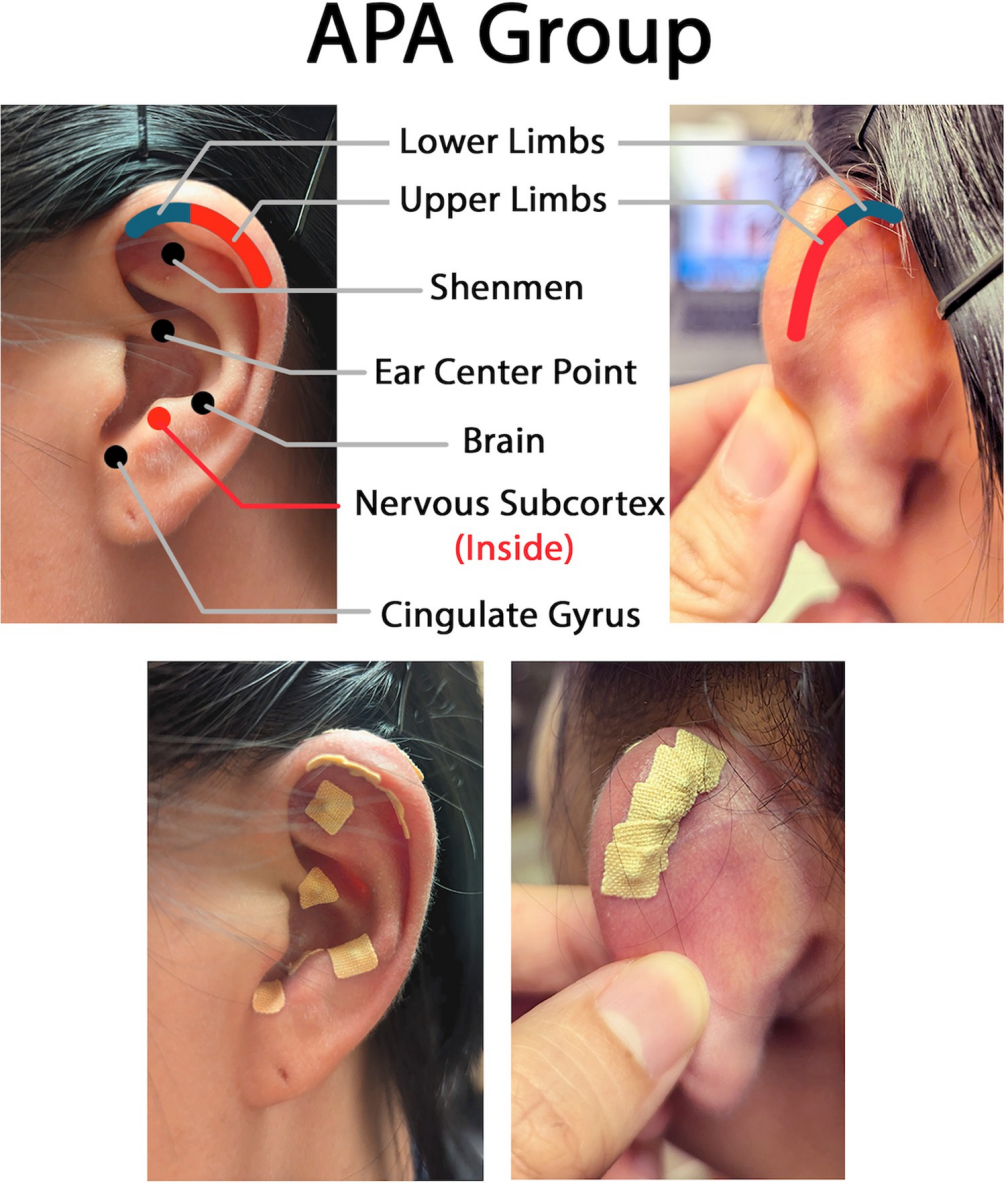
Ear points for chemotherapy-induced neuropathy. The figure shows ear points for chemotherapy-induced neuropathy based on the International Standards for Reporting Interventions in Clinical Trials of Acupuncture guidelines. This figure is original and licensed under CC BY 4.0. You may share and adapt it with proper attribution.

The APA provided in our study adheres to the guidelines set by the International Standards for Reporting Interventions in Clinical Trials of Acupuncture (STRICTA) [43]. The points for CIN include corresponding points located on the ears, at the front and back of the ear (depending on the body pain location), and five points known for alleviating stress and pain (i.e., *shenmen*, *ear center point*, *brain point*, *cingulate gyrus*, and *nervous subcortex*) (Fig 3).

#### Seed placement, treatment materials, dose, and stimulation of points

**Fig 4:**
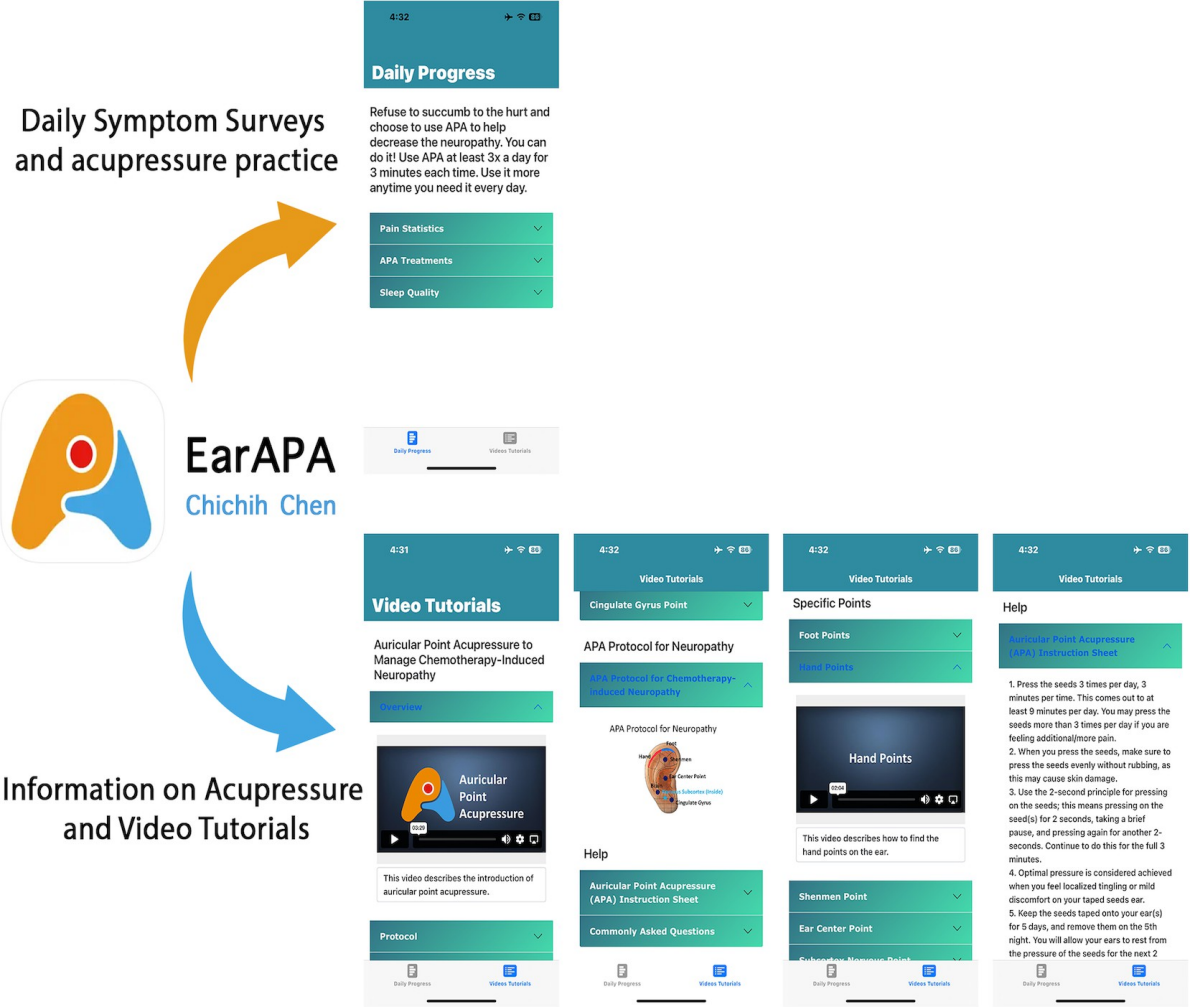
EarAPA smartphone application. This is the smartphone application through which the participants receive educational information on seed placement and stimulation.

Trained interventionists would apply the seeds to the ear points and instruct the participants to identify the ear points. The participants would be instructed to clean the outer ears and ear lobes with a 75% alcohol pad before placing pre-prepared tape with seeds on both ears. Preparing, cleaning, locating the ear points, and placing the seed procedures last 5 to 10 min. Participants are advised to sit quietly in comfortable chairs throughout the process. Participants in the sham control group receive seed placement and stimulation guidance via a smartphone application called “EarAPA” (Fig 4) and are asked to follow the instructions.

*Treatment materials*. *Vaccaria* seeds, which are natural, non-toxic botanical seeds of no medical value, approximately 2 mm in diameter, are used along with waterproof tape, each about 6 mm^2^ in size. The waterproof tape covering the seeds can safely stay on the ear for up to 5 days. Participants can shower and wash their hair during the intervention without affecting the seeds/tapes.

*Treatment dose*. The APA treatment duration includes four seed placement sessions with weekly cycles [15, 41] and daily stimulation of the ear points.

*Stimulation of ear points*. After applying seeds to specific points on the ear, participants are instructed to apply pressure on the seeds through the tape covering each ear point evenly, for 3 minutes at a time, 3 times a day, for a minimum of 9 minutes daily, regardless of whether they are experiencing clinical pain. Avoiding rubbing is essential to prevent skin damage and displacement of the seeds. Participants should also include a brief 2-second pause between each pressing.

The optimal pressure is reached when the participant experiences localized tenderness or slight discomfort. At the end of the 5^th^ day, participants are instructed to remove all tapes and seeds. The interventionist demonstrates the pressing technique to the participants, emphasizing steady pressure until they feel slight discomfort or tenderness. If any adverse effects occur, patients are advised to immediately contact the study center and return for evaluation, adjustments, or possible tape removal.

#### Comparison groups

*In-person APA (mAPA) group*. Participants in this group receive one in-person seed placement, in-person training for the participant or caregiver to place the seeds, and one secure Zoom session for APA coaching.

*Sham control (Virtual APA*: *vAPA) group*. Participants in this group are asked to review and follow instructional videos on the APA smartphone application on seed placement and stimulation and receive one Zoom session for APA coaching one week after the baseline study visit.

*Usual control group*. Participants continue with their usual care and are re-randomized into one of the APA intervention groups after the 4th-week visit (T2).

### Interventionist training and fidelity

The training protocol used in our studies (funded by the American Cancer Society [40] and R01 [44]) is revised and employed in this study to teach seed placement to the interventionists. The fidelity of the interventionists is ensured and assessed through several measures. Firstly, interventionists demonstrate proficiency by completing written and oral examinations. During the practice sessions, the interventionists are observed and mentored. Additionally, with the consent of participants, interventionists send the principal investigator (PI) a photo of their seed placement on the ears for assessment; these images are reviewed during debriefing meetings and on a regular bi-monthly basis. The adherence script is audiotaped and monitored to avoid bias and maintain the blinding of participants. Furthermore, inter-rater reliability in identifying ear points between the PI and the interventionist is established. If the Kappa scores fall below 0.8, additional training is provided to improve reliability.

### Randomization

After collecting pre-intervention data, all eligible participants are randomly assigned to one of three groups. The systems analyst and statistician use statistical software with a random number generator for the randomization process. Before recruitment begins, the software generates a list of group assignments to ensure real-time randomization immediately following pre-intervention assessments. Randomization occurs in blocks of three or six participants, distributing them evenly among the three groups based on the anticipated number of eligible participants. The software selects the next available record in the randomization list and assigns the participant to the corresponding group accordingly.

### Blinding

Due to the nature of the proposed intervention, we are unable to mask participants who are randomly assigned to the Control Group. To prevent bias stemming from perceived treatment effects or intelligent guessing, participants in both the APA Group (mAPA) and Sham control group (vAPA) are kept unaware of their group assignment. While the interventionists are blinded to the group assignments, they adhere to a scripted interaction with the participants. These interactions are audio-recorded for regular monitoring to ensure consistency. The group assignments will be blinded to the PI and Co-Is. The PI and Co-Is are not allowed to directly engage with the participants during the intervention or outcome assessments. Additionally, data collectors for outcome assessments are unaware of group assignments since no seeds are placed on the participant’s ears during data collection. Only a select few study personnel are privy to group allocations.

### Outcome measures

Table 2 lists the summary of measures where all measures can be completed within 70–80 minutes.

**Table 2:** Summary of study measures and time to complete all measures/study visit.

Construct/ Specific Measure	Items	Time (min)
**Self-reported Questionnaires**		**20–30**
CIN Symptoms (Revised BPI-CIN and CTCAE)	16+2	
Physical Function: Revised BPI-CIN	7	
Quality of Life: PROMIS-29	29	
Opioid Use (Name and dosage)	2	
Demographics Questionnaire (T1 only)	19	
**Objective Measures**		**50**
Sensation and Threshold	** **	25
**•** Quantitative Sensory Test Battery		
**•** Grooved Pegboard Test		
Endogenous biomarkers (Blood draw)		5
Neuroimaging (fMRI)		20
EMA (Daily CIN symptoms, APA practice)		1–3
Total Time (Excluding EMA)		70–80

Note. Chemotherapy-induced neuropathy, CIN; Brief Pain Inventory-Chemotherapy Induced Neuropathy, BPI-CIN; Common Terminology Criteria for Adverse Events, CTCAE; Patient-reported Outcomes Measurement Information System, PROMIS; functional magnetic resonance imaging, fMRI; Ecological momentary assessment, EMA; Auricular point acupressure; APA, Auricular point acupressure

#### Primary outcomes

The study’s primary outcomes are the mean change of CIN symptoms, and measured by the revised BPI-CIN (pain, numbness, or tingling) [17] and the Common Terminology Criteria for Adverse Events (CTCAE) version 4. The physical function is assessed using the subscale of BPI-CIN pain interferences [17].

#### Secondary outcomes

The secondary outcomes are changes in pain sensory and thresholds, motor and cognitive functions, inflammatory signaling, brain connectivity, opioid use, and quality of life. Pain sensory and thresholds are measured using the QST battery, which includes light touch sensation (SWMT), threshold and tolerance, temporal summation, and conditioned pain modulation (CPM). Threshold responses are conducted in randomized and counterbalanced order, with CPM always occurring last. Temporal summation measures, CPM, and after-sensation responses to these measures are combined to create the central sensitization index for use in statistical analyses [45].

Motor and cognitive functions are assessed using the Grooved Pegboard Test (GPT), which measures fine motor skills, cognitive processing speed, sensorimotor function, and manual dexterity [46]. The test consists of a 5-by-5 slotted board with a series of small, randomly arranged holes and a set of pegs with grooves that match the contours of the holes. Participants are required to insert the pegs into the holes as quickly as possible, and the pegs have to be rotated to align with the grooves while being inserted.

Inflammatory signaling is evaluated by measuring endogenous inflammatory biomarkers (a panel of cytokines and chemokines) from peripheral blood samples using the bio-plex pro-human chemokine panel. All specimens are transported in appropriate containers with dry ice, then multiplexed and duplicated in assays. Analysis is conducted using Bio-Plex Manager software at an Immune Monitoring Core at an Oncology Center, with a technician, unaware of the data collection time points, performing the analysis. The levels of plasma markers are measured using a multiplex bead-based immunofluorescence assay (Luminex-200 system, Luminex, Austin, TX). This includes IL-1α, IL-1β, IL-2, IL-4, IL-6, IL-8, IL-10, IL-12, IL-13, IL-17, IFN-γ, TNF-α, CGRP, MCP-1, Eotaxin, CRP, and TGF-β. Sample concentrations are calculated using a five-parameter regression formula based on the standard curves. To ensure accuracy, biomarker quantification will be conducted in duplicate after the data collection is completed.

Brain connectivity is assessed through neuroimaging using functional Magnetic Resonance Imaging (fMRI), performed on a 3.0 Tesla Siemens Prisma System (Siemens Medical Solutions, Erlangen, Germany) at a radiology unit in Baltimore, Maryland. Blood Oxygen Level Dependent (BOLD) functional images are acquired using 2D gradient-echo echo-planar imaging, covering the entire head (TR = 2000 ms, TE = 30 ms, flip angle 90 degrees, acquisition matrix 64x64x40, slice thickness 4 mm). For each fMRI data point, 300 volumes are acquired [47]. All imaging systems are integrated with the hospital’s picture archiving and communication system. Each scan session lasts approximately 20 minutes.

Opioid use is measured through an EarAPA smartphone application during the four-week program [48]. This ecological momentary assessment (EMA) app is programmed to deliver 2 daily prompts to ask participants to complete a short survey (1–2 min) for medication use. The dose of morphine equivalent (Milligram Morphine Equivalent, MME) is calculated by using an equivalency factor to determine the equivalent dose of the prescribed opioid. Daily *morphine equivalent dosing* is the sum of the MME of all opioids a patient takes within 24 hours and is calculated to MME for analysis [49].

Participants’ quality of life is measured using the self-report Patient-Reported Outcomes Measurement Information System (PROMIS) 29-item Adult Profile version 2. Developed by the National Institutes of Health (NIH), this comprehensive measure assesses multiple aspects of health-related quality of life, including physical function, pain intensity, fatigue, emotional distress, and social health [50].

### Study procedures

Research staff conduct a phone screening interview with potential participants to determine their eligibility and willingness to participate in the study. Subsequently, a baseline study visit is scheduled to discuss the study in detail, obtain written informed consent, and complete baseline assessments, which include completing a REDCap survey, Grooved Pegboard test, QST, and a peripheral blood draw. All participants are asked to set up an EarAPA application on their smartphone devices. This application contains instructional and tutorial videos on APA intervention. During the 4-week program, the application sends a daily notification to ask participants to complete the short survey to measure real-time CIN symptoms, adherence to APA, and opioid use.

Participants are randomly assigned into three groups: (1) an in-person treatment and training on administering APA (mAPA) group, (2) a sham control (vAPA) group that receives instructions to follow instructional videos on administering APA, and (3) a Wait-List Usual Care Control (UC) group. One week after the first APA treatment, participants in the mAPA and vAPA groups attend one Zoom meeting to receive coaching on seed placement. Study outcomes are collected at baseline (pre-intervention: T1), post-completion of the 4-week APA treatment (post-intervention: T2), and at monthly follow-ups for three months post-completion of treatment (M1-M3).

For the fMRI component, participants who agree to participate in the study are asked for their willingness to undergo an fMRI. Additional screening is performed to ensure the safety of the fMRI procedure. The fMRI is performed immediately following the assessment for the baseline visit (T1), immediately post-intervention (T2), and at the one-month follow-up (M1). Based on our pilot studies, the overall study visit—including all assessments, interventions, and fMRI—can be completed within 2 hours.

### Safety considerations

The study includes various procedures, each carrying potential risks necessitating consideration and mitigation.

#### Auricular point acupressure (APA) intervention

Patients may initially experience discomfort or pain at the ear points, which typically subsides within days. Infrequent risks include skin irritation, allergic reactions to tapes, bruising, and bleeding at acupressure points due to excessive pressure. Rare risks entail infection or accidental ear seed insertion into the ear canal. Proper training and participant instructions will help alleviate these risks.

#### Blood draw

Participants may experience minor discomfort, soreness, bruising, bleeding, or needle-related anxiety. Rare risks include infection at the insertion site and fainting.

#### fMRI scan

Magnetic resonance imaging (MRI) presents physical risks due to strong magnetic fields, potentially leading to tissue disruption. Participants with metallic implants will be screened and excluded. Infrequent risks include dizziness, nausea, headache, or discomfort during the scan, which can be minimized by remaining still. Other risks involve physical discomfort while lying in the MRI, alleviated with pillows and blankets. Participants will have a visual screen to mitigate discomfort.

#### Quantitative sensory testing

Thermal Pain testing involves applying heat to the skin (up to 122°F or 50°C) with minimal burn risk due to safety features in the Medoc device, comparable to common household appliances. Previous studies using similar procedures found no serious adverse effects. Punctuate Probe Pain Testing carries a slight risk of skin puncture, particularly for those with thin skin, while Cold Water Testing presents no significant risks from hand immersion. Participants are informed of their ability to stop testing at any time.

#### Steps to minimize risks

Potential participants will undergo comprehensive informed consent procedures before participation, ensuring understanding and voluntary involvement. Project staff will be trained to conduct the informed consent process. Participants will be informed of their right to withdraw without penalty. Strict confidentiality measures, including data coding and secure storage, are implemented.

#### Plan for reporting unanticipated problems or study deviations

All staff members are responsible for monitoring, detecting, and documenting any adverse events that occur during or after the intervention. This includes evaluating potential adverse events such as pain, bleeding, bruising, skin irritation, displacement of seeds, allergic reactions, dizziness, nausea, vomiting, or fainting. The events will be reported to the Principal Investigator (PI) and categorized by severity, frequency, relationship to the study product, and outcome (1 = recovered/resolved; 2 = ongoing; 3 = recovered with sequelae; 4 = fatal; 5 = unknown). Serious events requiring medical intervention or resulting in significant outcomes will be reported immediately and in accordance with the Institutional Review Board (IRB) policy on reportable events. Reports are securely stored for reference.

### Data management and analysis

We incorporate techniques from a previously approved trial on APA intervention conducted by our study team to optimize data handling and analysis in the current study [51]. For this study, a digital data entry system with password protection facilitates direct entry into the database. Electronic forms with customized data entry are employed for all clinic visits. iPads equipped with a direct data entry system are provided for study participants and coordinators. The PI maintains oversight of data management. Each participant is assigned a unique study identifier, used consistently across all measures, documents, and files in statistical analysis and manuscript preparation.

Personal identifiable information required for informed consent tracking is securely stored separately from other data and accessed solely by authorized team members. Participant information remains confidential, and personal data is not released. Plasma samples for the biomarkers study are labeled with the study ID and collection date before being stored at -80°C in a password-locked freezer and laboratory.

#### Treatment of missing data

By utilizing direct data entry, we aim to reduce the occurrence of missing data. In cases of study drop-outs, we investigate the reasons behind the drop-outs to assess the missing data mechanism. If the missing data conforms to the assumption of missing-at-random (MAR), likelihood-based methods that ignore the response mechanism will be used [52]. Conversely, if missing data depends on unobserved data even after conditioning on observed data (i.e., non-ignorable missing data), selection and pattern-mixture models will be considered to inform our chosen model [52]. Sensitivity analyses will be conducted to evaluate the impact of inferences of missing mechanism assumptions [53].

#### Statistical analysis

The intention-to-treat analysis (ITT) will be the primary analysis method for this study and will be conducted for all participants according to their original randomization. Summary tables will be provided for all variables. To determine the efficacy of APA on CIN symptoms and physical function after a 4-week APA program, we will use a generalized estimating equations (GEE) approach to examine differences in the change in outcomes from pre- to immediate post-intervention among the three groups. The group-by-time interaction will be the main parameter of interest. Variables to be entered into the model will include demographic factors (age, ethnicity, and sex) and the use of other therapies as covariates in the final analysis.

In addition to reporting the mean score changes of outcomes, we will use a cut-point of 30% improvement [54, 55] for responder analysis. For responder analysis of the primary outcomes, we will use the GEE model [56] described above, changing it to a logistic model. In both models, violation of assumptions will be detected by corresponding model diagnostics [57].

Using the approach outlined above, we will conduct additional analyses at 1, 2, and 3 months to explore the maintenance of the APA effect over time. To examine the influence of other therapies that participants may have used, the dose (frequency x duration) of each therapy will be included in the analytical model as covariates in a sensitivity analysis to determine whether the effect of the intervention remains after controlling for other therapies.

*Analysis plan for neuroimaging*. Following standard processing for fMRI data (to include slice timing correction, motion correction, artifact removal, nuisance regression, temporal filtering, normalization, and spatial smoothing), 6 mm regions of interest will be placed on target networks, from which the eigenvariate of the BOLD signal time courses will be extracted. Pearson’s correlation coefficients will be calculated for each ROI pair, and z-scores will be normalized for between-subject comparisons for the networks between the scans of different imaging groups (T1-M1). The GEE models described above will be used to examine group differences in change from pre- to follow-ups and will be performed to assess for significant differences in correlations.

### Data safety and monitoring

The study emphasizes participant safety through regular monitoring of data quality, safety, and adherence to protocols by the Principal Investigator (PI) and study staff. Meetings are scheduled weekly with the project coordinator and monthly with the investigative team to ensure study progress. A Data and Safety Monitoring Report is submitted annually to the Institutional Review Board (IRB), and any adverse events are reported immediately. A Data Safety and Monitoring Board (DSMB) is established to review study progress and safety measures every six months, with the authority to recommend whether the study should continue. Costs of participation are not charged to participants, and the study aims to provide innovative approaches to managing chemotherapy-induced neuropathy (CIN) through auricular acupressure (APA). Stopping rules are outlined to ensure participant safety, including discontinuation of intervention in case of adverse effects or recruitment challenges, with decisions made collectively by the PI, Study Monitoring Committee, and relevant governing bodies. Since this document is a study protocol, it does not contain any data and is in compliance with the data policy of PLOS.

## Discussion

The proposed study aims to further investigate the efficacy and mechanisms underlying APA for CIN management, as CIN is a prevalent side effect of cancer treatment. With advancements in cancer treatments leading to improved survival rates, the long-term effects of CIN have become increasingly apparent, negatively impacting individual physical function and quality of life [58]. APA has emerged as a promising intervention for CIN, building on the theory of auricular acupuncture developed by Paul Nogier in the 1980s.

Our pilot data on APA for CIN demonstrated significant reductions in pain severity, numbness, and tingling, along with improvements in sensory nerve function and pain modulation [18, 19, 59]. Functional MRI data revealed immediate and sustained changes in brain connectivity following APA, indicating its potential to modulate the neuronal pathways associated with pain perception and inflammation [18].

In this paper, we describe a rigorously designed study to investigate APA efficacy in managing CIN, while also examining the underlying mechanisms that contribute to its effectiveness. Through a randomized controlled trial, we assess the effects of in-person and virtual APA interventions compared to usual care control. By employing rigorous outcome measures, including changes in CIN symptoms, sensory function, inflammatory biomarkers, brain connectivity, opioid use, and quality of life, we aim to comprehensively evaluate the impact of APA on multiple domains of CIN.

Guided by established theoretical frameworks and previous research findings, our study examines the complex interplay between inflammatory biomarkers, pain sensitivity, brain activity, and psychological factors in mediating the effects of APA on CIN. Furthermore, by exploring sustained effects up to three months post-intervention, we seek to provide insights into the long-term benefits of APA in managing CIN symptoms and improving functional outcomes.

We hope that this study will provide robust evidence of the efficacy of APA on pain and CIN symptoms and advance our comprehension of the underlying mechanisms behind its effectiveness. If proven to be effective, APA can be integrated into routine clinical practice to benefit cancer patients during treatments. Understanding the underlying mechanisms of its effectiveness will enable the refinement of more tailored and personalized APA interventions, optimizing outcomes for individual patients.

## Supporting information

S1 Checklist Standard protocol items: Recommendations for interventional trials [SPIRIT] 2013 checklist. (DOCX)

S1 File Full protocol. (PDF)
