# Supplementary material for: A randomized controlled study of auricular point acupressure to manage chemotherapy-induced neuropathy: Study protocol
Source: PLoS One. 2024 Sep 26;19(9):e0311135. doi: 10.1371/journal.pone.0311135 (PMC11426428; doi:10.1371/journal.pone.0311135)
Supplement: S1 File — (PDF) [file pone.0311135.s002.pdf]

JHM IRB - eForm A – Protocol

- **Use the section headings to write the JHM IRB eForm A, inserting the appropriate material in each. If a section is not applicable, leave heading in and insert N/A.**
- **When submitting JHM IRB eForm A (new or revised), enter the date submitted to the field at the top of JHM IRB eForm A.**

****

1. Abstract

Chemotherapy-induced neuropathy (CIN)—pain, numbness, or tingling distributed in the hands and feet—produces persistent symptoms affecting sensation and balance in cancer survivors[1-5]. Up to 50% of cancer survivors still suffer CIN 6 years after treatment[3,4]. Duloxetine, the only recommended drug by the American Society of Clinical Oncology[6], was found to be superior to placebo but improved CIN by only 0.73 points (0-10 scale)[7]. No effective treatment for CIN has been established except exercise, with an effect size of <0.50[8]. Opioids relieve CIN pain,[9,10] but long-term use is strongly discouraged due to opioid overuse[11].

We propose to test auricular point acupressure (APA), an innovative and scalable solution developed from auricular acupuncture. APA is a non-invasive (needleless) and active treatment for patients with pain, whereas acupuncture is an invasive (using needles) and passive treatment (administered by a licensed practitioner). In APA, small seeds are taped on specific ear points by a skilled provider and patients press on the seeds to stimulate ear points three times daily, three minutes per time, for a total of nine minutes per day. APA provides pain relief within 1–2 minutes after ear stimulation and sustains pain relief for one month after a 4-week APA intervention[12-20]. APA is popular in Taiwan, China, and Europe. Though its use is sparse in the U.S., a limited number of clinical trials have supported APA in pain management[21,22].

2. Objectives (include all primary and secondary objectives)

The proposed study will evaluate APA on CIN, rigorously considering point specificity and placebo effects by integrating self-report measures, psychophysical measures (QST), endogenous biomarkers (cytokines), and neuroimaging to investigate APA's efficacy and underlying mechanism(s). The data will be collected at pre-intervention (T1), post-completion of the 4-week APA treatment (T2), and follow-ups at monthly post-completion of treatment for 3 months (T3-T5), and follow-up data collection at 6, 9 and 12 months (T6–T8) for a total of 8 assessments. We will use a randomized control trial, three-group design: (1) In-person weekly treatments and self-guided smartphone application to understand and administer APA (mAPA), (2) A virtual APA (vAPA): mAPA + plus secure zoom sessions for APA coaching with questions and answers), (3) Wait-List Usual Care Control (UC): After one month follow-up, the participants will be re-randomized into (1) mAPA or vAPA group. A smartphone application for ecological momentary assessment (EMA) will be used to monitor APA adherence and capture momentary CIN severity and analgesic use.

Our specific aims include:

Aim 1: Determine the efficacy of APA for CIN at 1-month post-APA (**primary endpoint**) and explore monthly follow-ups to 3-months post-APA to assess sustained effects. ***H1:*** Participants in the APA Groups will experience significant CIN improvement (defined as a 30% change at 1 month post-APA)[1,2] in CIN severity and physical function (**primary outcomes**) compared to Waist-List Usual Care group.

Aim 2: Determine the effect of APA on pain and sensory thresholds, inflammatory signaling, brain connectivity, opioid use, and quality of life (**secondary outcomes**). ***H2:*** Participants in the APA Groups will

have improvement in pain and sensory thresholds, regulation of plasma inflammatory profile (increases in anti-inflammatory cytokines and decreases in pro-inflammatory cytokine), and change in brain functional connectivity compared to Wait-List Usual Care group. For patients on opioids, the APA Group will have a reduction in morphine equivalent daily use.

Aim 3: Examine the mechanisms underlying the beneficial/non-beneficial effects of APA on CIN. ***H3:*** APA-induced CIN reduction will be mediated by changes in inflammatory signaling profile, functional connectivity, and/or in cancer-related symptoms (e.g., fatigue and sleep) and moderated by nonspecific placebo effects (i.e., treatment expectation) and demographic factors (e.g., sex/gender and race/ethnicity).

3. Background (briefly describe pre-clinical and clinical data, current experience with procedures, drug or device, and any other relevant information to justify the research)

More than 60% of cancer patients experience chemotherapy-induced neuropathy (CIN), a severe side effect of chemotherapy (including platinum drugs, vinca alkaloids, taxanes and/or bortezomib)[4,25]. CIN is a predominantly sensory neuropathy (i.e., numbness, tingling, and pain) that may be accompanied by motor change (i.e., loss of strength)[4,26]. CIN may cause treatment delays, dose reductions, or discontinuation, which can affect survival rates[27]. With improved cancer treatments and longer survival, the late effects of CIN continue to produce a significant burden in up to 50% of cancer survivors who are suffering from CIN 6 years after treatment, with a 1.8-fold increased risk of falls[28]. CIN continues to cause significant functional disability, negatively impacts quality of life[3,4], and demands significantly high healthcare costs and resource use[27].

The pathophysiology of CIN remains unknown[25]. Hypotheses have been proposed including damage to mitochondria in peripheral sensory neurons[29] or inflammation[29,30]. The majority of CIN arises from damage to dorsal root ganglion neurons or their axons, leading to pain, sensory loss, and at times sensory ataxia[30], which might exacerbate symptoms of peripheral nerve damage[31]. The inflammatory component results from activation of microglia, astrocytes, and satellite glial cells in the dorsal horn of the spinal cord, which leads to the production and release of proalgesic mediators, such as tumor necrosis factor (TNF) and IL-1β[32,33]. As such, an effective CIN treatment should be able to regulate inflammatory biomarkers to achieve CIN relief.

Treatment for CIN is limited. No uniformly effective treatment for CIN has been established. *Pharmacotherapy* is the predominant treatment for CIN. Opioids do relieve neuropathic pain[9,10] but long-term use is strongly discouraged due to opioid overuse[11]. Gabapentin, a safer alternative to opioids, is a Food and Drug Administration approved first-line treatment for diabetic neuropathy but it is not effective for CIN[34-36], suggesting the mechanisms of CIN may differ from other forms of peripheral neuropathy. Duloxetine[37], the only recommended drug by the American Society of Clinical Oncology[6], was found to be superior to placebo but can improve CIN by only 0.73 points (0-10 scale)[7]. *Nonpharmacological therapy*, including exercise[38], acupuncture[39-41], scrambler therapy[42,43], electrical nerve stimulation[44], yoga[45], dietary supplements[46-48], or self-care strategies[49], have limited effects or barriers for universal success. Exercise is commonly suggested but remains burdensome[38] and the effect size of exercise to reduce CIN is less than 0.50, which cannot effectively manage CIN[8]. While acupuncture has shown promising CIN relief[39-41], its use is limited because of high dropout rates due to frequent office visits[50], limited insurance coverage[51], and fear of needles[52].

Cancer patients already receive a combination of drugs and are exposed to various adverse effects. Ideally, patients could be provided with non-pharmacological options to manage CIN, APA has the potential to provide rapid and low-cost relief without side effects, prolonged effort, and frequent provider-office visits.

Auricular point acupressure (APA) may be a solution to address current shortcomings of CIN treatment. APA, derived from auricular acupuncture/auricular therapy, was developed from Chinese medicine into modern science in the 1980s by Paul Nogier, MD[53-55]. Dr. Nogier mapped a somatotopic representation of the human body onto the ear. Specific points of the ear correspond to specific organs and areas of the body. By stimulating these ear points, symptomatic parts of the body can be treated. In Dr. Nogier's system of

diagnosis, the location of ear point corresponding to the symptomatic body is confirmed by electrodermal responses (i.e., an electrical point finder)[56,57]. Once identified, these points can be stimulated—classically with needles, electrically[57,58], or with APA[15]. The underlying theory of auricular acupuncture posits that nerves in the outer ear correspond to specific areas of the brain, and these areas have a reflex connection with specific parts of the body[57,58]. Correlation of ear points and brain activity has been validated by fMRI[59,60].

4. sIRB protocol

The lead/coordinating cancer (LCC) will be Johns Hopkins. Specially,

a) Dr. Nada Lukkahatai will serve as LCC PI and has contact information for all sites (Johns Hopkins University and University of Texas Health Science Center at Houston (UTHealth).
b) LCC PI (Dr. Lukkahatai) confirms that each participating center has on file an FWA with OHRP.
c) LCC PI (Dr. Lukkahatai) and site PIs (Dr. Constance Johnson at University of Texas Health Cizik School of Nursing) agreed to a sIRB to Johns Hopkins and all PIs reviewed all IRB documents. Site IRB will cede to this sIRB once approved.
d) All PIs will be responsible for the coordination and overall management of the project and its scientific integrity at their respective sites. They will hold weekly meetings via video and/or telephone conferences, in addition to biweekly (twice a week) email communications, sooner when needed. Once the protocol is approved, this will be the only document used by each PI in recruitment sites. Any amendments will be agreed by all PIs and will seek IRB approval prior to use. Such amendment will then be implemented by all PIs as the most current version.
e) All PIs will use Johns Hopkins RedCap for data collection and management to consolidate all data in one site. We will use this paperless data entry system with password-protected access to allow data to be entered directly into the REDCap to avoid missing and incorrect data entries. The database will be regularly backed up. Data will be collected using a REDCap link. The PIs will oversee all data collection and management for each site. All of the data will be encrypted and stored in a Microsoft SQL server and REDCap on the JHU project server with a date/time stamp. All participants will be assigned unique study identifiers that will appear on all data collection instruments, documents, and files used in statistical analyses. Personal information is needed only for tracking informed consent which will be stored separately from other data and accessible only to select or key team members. No participant identifying information will be released.
f) All PIs will oversee protocols on their research sites. Protocol training will be done with any research assistant involved in the study to ensure that the protocol is strictly followed. Any deviation will be reported to the PIs. The PIs will meet at least biweekly via any telecommunication session to discuss study implementation.

5. Study Procedures

a. Study design, including the sequence and timing of study procedures (distinguish research procedures from those that are part of routine care).

This prospective randomized controlled study will randomly assign participants into three groups: (1) In-person weekly treatments and self-guided smartphone application to understand and administer APA (mAPA), (2) A virtual APA (vAPA): mAPA + plus secure zoom sessions for APA coaching with questions and answers), (3) Wait-List Usual Care Control (UC). During intervention phase, participants in the mAPA Group will receive one in-person seed placement and a training for the participant or their caregiver to place the seeds on the ear points and one zoom meeting 1 week after the first visit to coach participant and/or caregiver on seed placement., Participants in vAPA will self-administer APA by pressing the seeds according to the video instruction. Participant and/or a caregiver will follow the video instruction on seed placement and receive one zoom session for APA coaching one week after the baseline visitwhile Usual Care Group will continue with their usual care. Participants in the Usual Care Control will have the opportunity to receive APA after completion of the study assessment. The EMA smartphone app will be

used to collect real-time CIN outcomes and adherence to APA practice for participants in the APA Group; participants in Usual Care Control will receive the app to measure real-time CIN outcomes only.

During the in-person study visits, the interaction between participant and interventionists will be audio-taped for regular monitoring to ensure consistency.

b. Study duration and number of study visits required of research participants.

This proposed trial will evaluate the APA sustained effects for CIN up to a 12-month follow-up. The data will be collected at pre-intervention (T1), post-completion of the 4-week APA treatment (T2), and follow-ups at monthly post-completion of treatment for 3 months (T3-T5), and follow-up data collection at 6, 9 and 12 months (T6–T8) for a total of 8 assessments. The primary endpoint is at 1-month follow-up. Sessions will be conducted starting between 9 and 11 am to control for circadian variation in cytokine levels[61,62]. For the fMRI component, we plan to recruit 36 participants to investigate the brain activity changes due to APA. Sessions will be conducted starting between 9 and 11 am to control for circadian variation in cytokine levels. APA visit will last ~30 minutes for first treatment, QST will last ~45 minutes, and fMRI will last ~45minutes.

c. Blinding, including justification for blinding or not blinding the trial, if applicable.

Participants cannot be masked to the Control Group to which they have been allocated because of the nature of the proposed intervention. Participants in the APA Groups will be blinded regarding group assignment and will be evaluated for the treatment allocation after the first APA treatment—not at the end of the completed treatments—to avoid bias due to the perceived treatment effects for intelligent guessing. The interventionists will not be blinded; however, they will follow a script when interacting with the participants and the interaction will be audio-taped for regular monitoring to ensure consistency. The PI and Co-Is will be blinded regarding group assignment and will not contact or interact with the participants during the intervention and outcome assessments. Data collector for outcome assessments will be blinded since there will be no seeds placed on the ears when the data are collected. Group allocation will be known by a minimum number of study personnel.

d. Justification of why participants will not receive routine care or will have current therapy stopped.

N/A. Participants will be able to receive routine care.

e. Definition of treatment failure or participant removal criteria.

Participants failing to continue to meet any of the inclusion/exclusion criteria will be removed from the study and an appropriate clinical referral provided. For example, the participation of participants developing a serious psychiatric disorder or symptoms requiring treatment (e.g., mania, suicidality, and psychosis), major medical illnesses or serious injuries, which would preclude full participation, will be suspended until they are stable and can continue participation.

f. Description of what happens to participants receiving therapy when study ends or if a participant's participation in the study ends prematurely.

N/A

6. Inclusion/Exclusion Criteria

Inclusion criteria. Eligible participants will meet the following criteria: (1) cancer patients ages ≥18 years, (2) have received a medication in one of the following categories: platinum-based, vinca alkaloids, bortezomib, eribulin, and/or taxanes, (3) have CIN due to receiving neurotoxic chemotherapy for cancer or

have pre-existing peripheral neuropathy of another etiology that worsened after chemotherapy for at least 6 months and (4) have one of the average intensity of pain, or numbness, or tingling on their extremities the previous week due to CIN ≥ 4 on a 11-point numerical scale.

Exclusion criteria. Participants reporting the following will not be eligible: (1) use of an investigational agent for pain control concurrently or within the past 30 days; (2) use of an implantable drug delivery system, e.g. Medtronic SynchroMed®; (3) prior celiac plexus block or other neurolytic pain control treatment; (4) other identified causes of painful paresthesia existing prior to chemotherapy (e.g., radiation or malignant plexopathy, lumbar or cervical radiculopathy,); and (5) allergy to latex (the tapes for the APA include latex) and/or having a history of allergic reactions to the adhesive tape.

7. Drugs/ Substances/ Devices

a. N/A The rationale for choosing the drug and dose or for choosing the device to be used.

N/A.

b. Justification and safety information if FDA approved drugs will be administered for non-FDA approved indications or if doses or routes of administration or participant populations are changed.

N/A.

c. Justification and safety information if non-FDA approved drugs without an IND will be administered.

N/A.

8. Study Statistics

a. Primary outcome variable.

Clinical Outcomes. CIN symptoms will be measured by: (1) Revised BPI-CIN (pain, numbness, or tingling)[12] (2) Common Terminology Criteria for Adverse Events (CTCAE) version 4 to assess the loss of sensory and motor (0–4 grading scale). Physical function will be assessed using the subscale of BPI-CIN pain interferences[12] and Eastern Cooperative Oncology Group (ECOG) performance status scale. Other measures include PROMIS 29, to assess QOL[75,76], Quick DASH index, MD Anderson Symptom inventoryto assess symptoms profile, pain impact, pain-self efficacy and pain castastrophizing score to measure the psychological impact of pain and demographics survey, Chronic Overlapping Pain Condition and Charlson Comorbidity Index to assess demographic and clinical conditions. Opioid use will be collected via EMA diary during intervention and T1-T8, using a questionnaire. Milligram Morphine Equivalent (MME) will be determined by using an equivalency factor to calculate a dose of morphine equivalent to the ordered opioid. Daily *morphine equivalent dosing* is sum of the MME of all opioids a patient is likely to take within 24 hours, and will be calculated to MME for analysis[77]. REDCap will be used to record and store clinical outcome measures.

b. Secondary outcome variables.

Sensation and Threshold. Dr. Campbell's lab has been conducting QST in chronic pain for over 12 years and will provide training and supervision on QST assessment. The QST battery consists of light touch sensation (SWMT), threshold and tolerance, temporal summation, and conditioned pain modulation (CPM). The threshold responses will be conducted in randomized and counterbalanced order; CPM will always occur last. Temporal summation measures, CPM, and after-sensation responses to these measures will be combined to create the central sensitization index for use in statistical analyses[78]. REDCap will be used to record and store sensation and threshold data.

Motor and cognitive functioning. The Grooved Pegboard Test (GPT) will be used to measure fine motor skills, and cognitive processing speed. The test consists of a board with a series of small, randomly arranged holes and a set of pegs with grooves that match the contours of the holes. Participants will be

required to insert the pegs into the holes as quickly as possible, and the pegs have to be rotated to align with the grooves while being inserted.

Neuroimaging. All fMRIs will be acquired on a 3.0 Tesla Siemens Prisma System (Siemens Medical Solutions, Erlangen, Germany) at The Johns Hopkins Hospital Radiology Building, Baltimore. Blood Oxygen Level Dependent functional images will be acquired using 2D gradient echo echo-planar imaging to cover the whole head (TR=2000ms, TE=30ms, flip angle 90 degrees, acquisition matrix 64x64x40, slice thickness 4mm); 300 volumes will be acquired for each fMRI data point[79,80]. All imaging systems are connected to the hospital picture archiving and communication system. Each scan will take ~20 minutes.

Endogenous Biomarkers. We are familiar with and have repeatedly tested cytokine biomarkers in our studies[81]. A 15-mL blood sample will be drawn at each time point and blood samples will be collected using standard phlebotomy procedures and will be transferred and processed at the JHSON basic science laboratory (coagulation and serum separation by centrifugation). Serum will be stored at -80 °C at the JHSON. Once the study collection is completed, a panel of cytokines and chemokines will be measured in diluted samples using the bio-plex pro-human chemokine panel. All specimens will be transported using an appropriate box containing dry ice and will be multiplexed and duplicated in assay and analyzed using Bio-Plex Manager software in the Immune Monitoring Core at the Johns Hopkins Oncology Center by a blinded technician who was not aware of the data collection time points. The plasma levels of IL-1α, IL-1β, IL-2, IL-4, IL-6, IL-8, IL-10, IL-12, IL-13, IL-17, IFN-γ, TNF-α, CGRP, MCP-1, Eotaxin, CRP, and TGF-β will be measured using a multiplex bead-based immunofluorescence assay (Luminex-200 system, Luminex, Austin, TX). A five-parameter regression formula will be used to calculate the sample concentrations from the standard curves. The quantification of biomarkers will be performed in duplicate to verify the results.

c. Statistical plan including sample size justification and interim data analysis.

Analysis Plan for Aim 1. The purpose of this aim is to determine the efficacy of APA for CIN (the highest score of pain, or numbness, or tinging) at 1-month post 4-weeks APA on primary and secondary outcomes. For continuous outcomes, we will use a generalized estimating equations (GEE) approach to examine differences in the change in outcomes from pre- to one-month follow-up among the three groups. The group-by-time interaction is the main parameter of interest. Variables to be entered into the model include demographic factors (age, ethnicity, and sex) and use of other therapies as covariate in final analysis. In addition to reporting mean score changes of outcomes, we will use a cut point of 30% improvement[23,24] for responder analysis. For responder analysis of primary outcomes, we will use the GEE model[82] described above, changing it to a logistic model. In both models, violation of assumptions will be detected by corresponding model diagnostics[83,84]. Using the approach outlined above, additional analyses will be conducted at 1, 2, and 3 months and at 6,9 and 12 months to explore the maintenance of the APA effect over time. To examine the influence of other therapies participants may have used, the dose (frequency x duration) of each therapy will be included in the analytical model as covariates in a sensitivity analysis to determine if the effect of the intervention remains after controlling for other therapies. OneDrive and SAFEdesk will be used to store data on-site and complete data analysis on-site or remotely, if needed.

Analysis Plan for Aim 2. The analytical strategies for Specific Aim 1 will be used to separately evaluate the effect of APA on biomarkers, sensitivity index, brain activity change, analgesic use, and QOL. Outcomes at one-month post-intervention will be examined using each biomarker individually and two latent variables with pre-defined biomarkers within the groups of pro- and anti-inflammatory biomarkers. All QST measures will have a separate z-score, which will be revised where appropriate, and combined to create the sensitivity index in statistical analyses[78]. We hypothesize that the analgesic effects of APA are the aggregated and counter-interacting effects of different biomarkers within each latent variable. Before analysis, we will check the normality assumption in the original data and log-transformed data. If normality cannot be met in both types of data, we will use two alternative approaches. We will apply a non-parametric test (e.g., Kruskal-Wallis test) to do multiple comparisons in each time difference among three groups; we

will categorize the change of biomarker measurement into increase (recoded as 1) and decrease (recoded as 0) from baseline, and apply a GEE, which can handle repeated measurements and subject heterogeneity. OneDrive and SAFEdesk will be used to store data on-site and complete data analysis on-site or remotely, if needed.

Analysis Plan for Neuroimaging. Following standard processing for fMRI data (to include slice timing correction, motion correction, artifact removal, nuisance regression, temporal filtering, normalization, and spatial smoothing), 6mm regions of interest will be placed on target networks, from which the eigenvariate of the BOLD signal time courses will be extracted. Pearson's correlation coefficients will be calculated for each ROI pair, and z-scores normalized for between-subject comparisons for the networks between the scans different imaging groups (T1-T5). GEE models described above will be used to examine group difference on change from pre- to follow-ups and will be performed to assess for significant differences in correlations.

Analysis Plan for Aim 3. This aim will examine the mechanisms underlying the beneficial/ non-beneficial effects of APA on CIN. Latent variable models will be used to examine the mediation effect of biomarkers, sensitivity index, and brain activity change.[85] Group assignment will be the independent variable and change in outcome from baseline to 1-month post-intervention will be the dependent variable. This model will adjust for fixed effects from important demographic and clinical covariates and variables related to missing data, if needed. Changes in inflammatory signaling profile, functional connectivity, sensitivity index, pain threshold, and cancer-related symptoms will be mediator variables. The parameter of interest will be the indirect effect of treatment group through mediators on changes in outcomes. Both the full mediation and partial mediation models will be tested. Treatment belief and expectations will be tested as moderators of the effect of APA on primary outcomes by entering the group by treatment belief into the GEE models described above. OneDrive and SAFEdesk will be used to store data on-site and complete data analysis on-site or remotely, if needed.

Analysis Plan for EMA data. Data from the smartphone EMA app will be analyzed per our published methods[86] and the following analysis plan, including daily adherence to APA practice, analgesic use, and real-time CIN outcomes (i.e., pain, numbness, tingling, and function). We will use mixed models and GEE[87] to test the relationship between adherence and changes in the primary outcomes. GEE, a multivariate extension of generalized linear models, accounts for dependence of responses within individuals due to repeated measures. The model will include adherence to APA practice, analgesic use, group, and the group-by-adherence interaction as predictors of daily levels of CIN outcomes over the entire EMA data collection period. The interaction is the parameter of interest because a higher adherence to APA should have a positive effect on pain. We will use the same approach to test for differences in analgesic use between groups. Models will use an autoregressive error structure to account for within-person correlations. Robust versions of GEE are available to handle longitudinal missing data[88]. We will test for missing data assumptions and utilize multiple imputation or full information maximum likelihood estimation to fill in missing observations. OneDrive and SAFEdesk will be used to store data on-site and complete data analysis on-site or remotely, if needed.

d. Early stopping rules.

We do not expect any adverse events related to the testing procedures as they have no remarkable health risks. However, we will report all adverse events and evaluate on a case-by-case basis whether the occurrence of a particular adverse event has implications for discontinuing the study to ensure participant safety and well-being.

Patients are allowed to drop out from the study if they do not feel comfortable with the procedures or they have an allergic reaction to the tape used for APA. We will conduct a safety interim analysis using the first 30 patients randomized (equally from all groups) comparing adverse reactions, pain, and analgesic use between the three arms at week 2 of the intervention and again at week 4. The safety outcomes will be reviewed without statistical analysis after the first 30 patients recruited.

9. Risks

a. Medical risks, listing all procedures, their major and minor risks and expected frequency. Listed below are all major procedures and associated risk.

Auricular point acupressure

Common Risk: Pain and discomfort on the ear points area. Patients' ear may feel discomfort when the seeds first apply but will diminish after a couple of days.

Infrequent Risks: Although infrequent, individuals may experience skin irritation or allergic reactions (redness, itching, swelling around the points) to the tapes and materials used in the APA, bruising and bleeding on the acupressure points due to excessive pressure and improper technique. The investigator and research team members will check with participants frequently to determine if they are experiencing any negative sensations. Participants will be screened for 1) history of allergic reaction to tapes and latex, 2) history of abnormal bleeding and excluded. If these symptoms occurred after participation, paticipants will be informed that for the participants' safety, they can no longer participant in the study.

Rare (Occurs in less than 1% or less than 1 out of 100 people): infection at the acupressure site, ear seeds accidentally fall into the ear canal. Although these risks are relatively low, all interventionists will be trained on proper auricular acupressure technique. Participants will be instructed to seek medical attention if they notice the issue.

Blood draw

Patient may feel minor discomfort at the injection site. Infrequent risk includes soreness to mild pain, bruise and/or bleeding at injection site; anxiety related to anticipation of needle stick; lightheadedness.
Rare (Occurs in less than 1% or less than 1 out of 100 people): infection at the insertion site, fainting.

fMRI scan

Common Risks: At each instance of the testing session there is a physical risk of doing an MRI scan unless precautions are taken before hand. In particular, MRI scans can be hazardous in the presence of some metallic devices, specifically: strong magnetic fields may dislodge metallic implants, causing bleeding and disruption of adjacent tissues. These fields may also cause erratic function of electrical pacemakers and stimulators. Radio waves may heat the body and metallic objects within or on the body, possibly resulting in burns. Certain metallic objects may move toward the magnet at very fast speeds if attracted by the magnetic field. Participants with any of these devices will be screened and excluded before hand.

Infrequent Risks: Although unlikely, they may also experience dizziness, nausea, headache, flashing lights, unusual tastes, numbness, or tingling while in the magnet or momentary loss of balance while leaving the magnet. These sensations are mostly due to movement while inside the magnet and can be minimized by holding still. All of these sensations should stop shortly after they leave the magnet. Additionally, because of the small space in the magnet, and the duration of the study, some people find the experiment to be uncomfortable or unpleasant. However, since the participants will have a visual screen to look at, they are unlikely to experience such feelings. Nonetheless, the investigator and the MR technician will check with them frequently to determine if they are experiencing any such negative sensations. They will be informed that they can discontinue the study at any time without penalty.

Other Risk: Although minimal, there is a small amount of physical discomfort while laying on one's back in the MRI. This will be alleviated by providing pillows and blankets to the participant. In addition, the cognitive task that we will give them might be challenging to some participants. They will be told that the task is designed to be challenging and that they shouldn't be disappointed by their performance during the testing session. There is also the possibility that we may detect something unusual or different on the MRI scans. The MRI scans in this study are done to answer research questions and not the type which reveal

medical conditions. In the unlikely event that we detect such abnormalities in the scan, the technologist will refer the scans to a qualified specialist (neurologist or neuroradiologist) for further examination and professional opinion as soon as possible after the scan. We will contact the participants by phone as soon as possible should the consulting specialist recommend further examination. The participants and their PCP will decide if they should have further examination or treatment. The consulting specialist will be available to answer any questions they or the PCP might have about the findings on the scan. The results of the research MRI scan will not become part of the participants hospital record.

Quantitative Sensory Testing:
Thermal Pain testing: There is a very small chance that the heating device might produce a burn, but the risks associated with the relatively small amount of heat applied to the skin (maximum of 122 degrees Fahrenheit or 50 degrees Celsius) are very low. The Medoc device has safety features built into both the hardware and software. The risks associated with use of this equipment are less than those associated with common household appliances. We have used the proposed laboratory pain procedures in a number of previous studies with healthy adults and chronic pain patients. No serious adverse effects were ever reported.
Punctuate Probe Pain Testing: There is a very slight chance that the probe might superficially puncture the skin for individuals with "thin skin."
Cold Water Testing: There are no significant risks from immersion of a hand in cold water. Again, participants are informed that they can stop testing whenever they would like to do so.

Questionnaires:
Minimal risks associated with completing questionnaires and diaries are subject fatigue and the possibility of minor psychological distress associated with answering sensitive questions regarding psychological functioning. Participants are permitted to omit answering questions they find distressing.

Sample and Data Analysis

At the end of the study specimen collection, all samples will be sent to UT Health, Cizik School of Nursing lab to be analyzed. None of the samples will be left in Johns Hopkins School of Nursing. All of Johns Hopkins enrolled participant samples will be sent out, an estimated total of 6,750 microcentrifuge tubes (225 participants * 5 times of blood collection * 6 microcentrifuge tubes each time). Once analyzed, the data will be sent to Dr. Constance M. Johnson at UT Health, Cizik School of Nursing through secure means (OneDrive). The lab at UT Health Houston will not retain any samples or data. Any unused samples will be destroyed and not stored or used for anything else. Both institutions will continue to collaborate after data and biospecimens transfer in form of joint analysis of data and publications.

The collected Redcap survey and EMA data will be transferred to the Co-PI, Dr. Constance M. Johnson at UT Health, Cizik School of Nursing for analysis. The data will be declassified and no person identifying information will be transferred. Secured, declassified participant survey responses will be transferred through OneDrive and /or Safe Desktop. A Johns Hopkins Data Trust Coordinator will be advised about the safest methods of data transfer.

Grant transferred to UT Health, Cizik School of Nursing makes UT Health, Cizik School of Nursing the primary award institute and Johns Hopkins School of Nursing will receive a subaward. This subaward will be used to continue recruitment and to follow participants till the end of study period. All participants recruited at Johns Hopkins School of Nursing are enrolled under the application IRB00243141, no participant was recruited under the initial application IRB00119665. As a multi-site study, other participants will be recruited at UTHealth with the same goal of jointly recruiting a total of 225 study participants. Data will be shared between Johns Hopkins School of Nursing and UTHealth, Cizik School of Nursing as UT Health, Cizik School of Nursing will also be recruiting participants into the study.

Participants were able to provide consent for biospecimen transfer under consent forms version 4 (discontinued) and version 1.12 (in use).

b. Steps taken to minimize the risks.

Potential participants will be fully informed of the purpose and activities involved in the research study during the screening process. Information from potential study participants that decide not to participate or are not eligible to participate will be deleted unless that individual consents to having their name, contact information, and general health information (e.g., diagnosis) kept in a study database for future study participation. Interested participants will be scheduled for an in-person visit, where written informed consent will be immediately obtained, prior to initiating any of the procedures. Project staff conducting informed consent will be appropriately trained by the PI, and will have completed formal coursework in the protection of human participants. The informed consent process will take place in a private location, and no time limits will be placed on the process. Potential participants will be encouraged to ask questions at any time. The protocol and informed consent forms will be approved by the IRB. Participants will be informed that they are free to discontinue participation in the study at any time, and that declining participation will in no way influence any current or future care they may receive. One copy of the signed consent form will be given to the patient and one will be kept in the study files for documentation.

All personnel involved in study procedures will be fully trained in the protocol. Regarding any discomfort that may result from completing study questionnaires, participants will be informed that they are free to refrain from answering any questions that make them uncomfortable or that they perceive as being particularly personal or sensitive. No participant will be considered ineligible due to declining to answer certain items or specific questionnaires. All pain testing equipment will be tested prior to use to ensure it is functioning properly. For all of the standardized laboratory pain procedures, participants are repeatedly informed that they may terminate any of the procedures at any time, and participants are monitored continuously by staff.

A number of procedures will be in place to prevent a breach of confidentiality from taking place. All potential participants will be fully informed of their rights pertaining to disclosure of personal health information (PHI) in accordance with HIPPA regulations. Confidentiality will be maintained by assigning participants a study number and numerically coding all data. One hard copy file linking the code number with identifying information will be kept in a separate locked file with direct access available to the PI and project staff only. All records and research data will be kept in locked filing cabinets or computers. Only summaries of group data will be reported in any publications or presentations, with no identification of individuals. These precautions should serve to minimize legal risks to participants.

MR Scanning: All participants are screened for contra-indications for magnetic resonance, namely severe claustrophobia, pregnancy, cardiac pacemaker, orthodontics (braces) or other non-MR compatible ferromagnetic implant. In addition, access to the scan room is strictly controlled to ensure that no ferromagnetic materials are introduced. The scanners to be used in this study are FDA approved and operates under radiofrequency power monitoring software at all times. All scan procedures at magnetic field strengths of 3T are FDA approved and are not classified as investigational devices.

The participants have the option of terminating the testing at any time without penalty if they so choose. The risks of psychological discomfort will be minimized by encouraging participants to let the examiner know if they are feeling any discomfort, and by continuous monitoring by experienced operators for subject fatigue, inattention, or annoyance. Scanning will be halted if the subject so requests, or if the examiner feels that the subject is becoming uncomfortable. The subject will be encouraged to report any adverse symptoms during the MRI. The MRI can be stopped by the subject either by verbalizing the request to stop

(which can be heard through the microphone in the MRI) or by pushing a "panic button" provided. Ear-plugs are provided to the participants to protect participants from scanner gradient noise.

A potential concern with MRI studies is so-called radiofrequency heating due to radiofrequency power deposition in the subject. The FDA has set strict and very conservative guidelines to guard against this risk. Such power deposition increases with the square of the magnetic field strength. However, it is possible to stay within these guidelines. To be 100% sure of compliance with the FDA guidelines, power deposition is monitored continuously by the software of the manufacturers and scans are not possible if the power deposition would exceed guidelines.

This study includes operator-controlled changes to the pattern and timing of an MRI signal or pulse sequence that varies from the standard sequences used by the MRI manufacturer at lower field strengths. These changes will operate within FDA guidelines.

c. Plan for reporting unanticipated problems or study deviations.

Any protocol deviations and/or adverse events (SAEs) will be systematically documented and reported immediately to the IRB. Adverse Event Reports and/or Case Report Forms will identify study participants by their initials and a unique study participant identifier to the JHM IRB Prompt Reporting Policy https://www.hopkinsmedicine.org/institutional_review_board/guidelines_policies/organization_policies/prompt_reporting_policy.html). If SAEs occur at the participants from local sites, site PIs must report SAEs to their local IRBs based on policy reporting requirements (https://www.uth.edu/cphs/policies/reporting-problems.htm for University of Texas Health Science Center at Houston or https://www.unlv.edu/sites/default/files/page_files/27/Research-RulesAndProceduresForConductingHumanSubjectsResearch.pdf for University of Nevada, Las Vegas. The participant's name will not be attached to any data. Copies of these forms will be stored in a locked office, which is only accessible to the PI and authorized personnel.

d. Legal risks such as the risks that would be associated with breach of confidentiality.

As detailed below, the investigators are quite careful regarding the protection of confidentiality, and multiple procedures are in place to reduce the likelihood of a breach of confidentiality. However, there is a small risk that information about participants could become known to people outside of this study, and this risk is identified in the informed consent form.

e. Financial risks to the participants.

There are no financial costs to study participants for any of the procedures.

10. Benefits

a. Description of the probable benefits for the participant and for society

While there are no likely direct and immediate benefits to the individual participants, subject's pain may relief if the intervention works.

Study participants will be paid for their time with care given to offering reasonable pay that is not excessive or coercive for those who are less advantaged. The goal of our study is to develop markers and assessment variables that will help to characterize risk for negative clinical outcomes following surgery. Enhancing our ability to improve clinical outcomes is likely to be viewed as an important result of the research. With the precautions and procedures to minimize risk as described in the previous section, we anticipate minimal complications and discomfort and believe the risk to benefit ratio to be favorable.

11. Payment and Remuneration

There is no pay to participate in this study. All participants will receive free parking, $40 for each visit to appreciate their time.

12. Costs

Detail costs of study procedure(s) or drug (s) or substance(s) to participants and identify who will pay for them. There is no cost to participate in this study. All participants will receive free parking, if applicable, free auricular point acupressure training, and a free acupressure kit.

13. Transfer of Materials

Transfer of biospecimens from Johns Hopkins to another organization for research purposes and receipt of biospecimens from an outside organization for your research must adhere to JHU policies for material transfer (https://ventures.jhu.edu/faculty-inventors/forms-policies/) and biospecimen transfer (https://hpo.johnshopkins.edu/enterprise/policies/176/39187/policy_39187.pdf?_=0.622324232879).

a. Will you **receive** biospecimens from an external entity for this research? [Yes/No].
No.

b. Will you **transfer** biospecimens to an external entity as part of this research? [Yes/No]
If "Yes", please address each of the following:
1) Describe the nature of the research collaboration with the external entity and the rationale for the transfer. (Include an explanation of your intellectual contribution to the design of the research study, resulting data and sharing, and participation in the planned publications.)
No.

2) Please confirm you will secure an MTA through the appropriate office (JHTV or ORA) prior to transfer.
(See: https://ventures.jhu.edu/technology-transfer/material-transfer-agreements/.)
No.

3) If the biospecimens you intend to transfer were obtained through clinical or research procedures at Johns Hopkins and "Other" is selected in Item 4, Section 23, please submit the following items in that Section:
No.

JHM IRB's role as sIRB:
Johns Hopkins Medicine is serving as the single IRB for this study. It is the preference of Johns Hopkins Medicine IRB to use the SMART IRB reliance agreement as the basis of reliance. The SMART IRB master reliance agreement was created in 2016 to harmonize and streamline the IRB review process for multisite studies. It enables reliance on a study-by-study basis, clearly defines roles and responsibilities of relying institutions and reviewing IRBs, and eliminates the need to sign reliance agreements for each study [e.g., a non-SMART IRB agreement]. 900+ institutions have already signed onto this agreement and are actively using it as the basis of reliance for multisite projects. Sites that will rely on JHM IRB are still responsible for conducting a local context review prior to the start of research at their site and for following any local and institutionally required policies as it applies to research at their site [e.g., reporting of unanticipated problems].

References

1. Shah A, Hoffman EM, Mauermann ML, et al. Incidence and disease burden of chemotherapy-induced peripheral neuropathy in a population-based cohort. *Journal of neurology, neurosurgery, and psychiatry.* 2018.
2. Park SB. Chemotherapy-induced peripheral neuropathy: highlighting unmet needs. *Journal of neurology, neurosurgery, and psychiatry.* 2018.
3. Staff NP, Grisold A, Grisold W, Windebank AJ. Chemotherapy-induced peripheral neuropathy: A current review. *Annals of neurology.* 2017;81(6):772-781.
4. Seretny M, Currie GL, Sena ES, et al. Incidence, prevalence, and predictors of chemotherapy-induced peripheral neuropathy: A systematic review and meta-analysis. *Pain.* 2014;155(12):2461-2470.
5. Miaskowski C, Mastick J, Paul SM, et al. Impact of chemotherapy-induced neurotoxicities on adult cancer survivors' symptom burden and quality of life. *Journal of cancer survivorship : research and practice.* 2017.
6. Hershman DL, Lacchetti C, Dworkin RH, et al. Prevention and management of chemotherapy-induced peripheral neuropathy in survivors of adult cancers: American Society of Clinical Oncology clinical practice guideline. *Journal of clinical oncology : official journal of the American Society of Clinical Oncology.* 2014;32(18):1941-1967.
7. Smith E, Pang H, Cirrincione C, et al. Effect of duloxetine on pain, function, and quality of life among patients with chemotherapy-induced painful peripheral neuropathy: a randomized clinical trial. *JAMA internal medicine.* 2013;309(13):1359-1367.
8. Kleckner IR, Kamen C, Gewandter JS, et al. Effects of exercise during chemotherapy on chemotherapy-induced peripheral neuropathy: a multicenter, randomized controlled trial. *Supportive care in cancer : official journal of the Multinational Association of Supportive Care in Cancer.* 2018;26(4):1019-1028.
9. Gilron I, Bailey JM, Tu D, Holden RR, Weaver DF, Houlden RL. Morphine, gabapentin, or their combination for neuropathic pain. *The New England journal of medicine.* 2005;352(13):1324-1334.
10. Gilron I, Tu D, Holden RR, Jackson AC, DuMerton-Shore D. Combination of morphine with nortriptyline for neuropathic pain. *Pain.* 2015;156(8):1440-1448.
11. Dowell D, Haegerich TM, Chou R. CDC Guideline for Prescribing Opioids for Chronic Pain--United States, 2016. *Jama.* 2016;315(15):1624-1645.
12. Yeh CH. L, N., Campbell, C., Sair. H., Zhang, F., Mensaha, S., Garry, C., Zeng, J., Chen, C., Pinedoa, M., Khoshnoodi, M., Smith, T.J., Saligan, L.N. Preliminary effectiveness of auricular point acupressure on chemotherapy-induced neuropathy: Part 1 self-reported outcomes. *Pain Mangement Nursing.* 2019;20(6):614-622.
13. Yeh CH, Lin WC, Suen LKP, et al. Auricular point acupressure to manage arthralgia related to aromatase inhibitors in breast cancer survivors. *Oncology Nursing Forum.* 2017;44(4):476-487.
14. Yeh CH, Suen LKP, Chien LC, et al. Day-to-day changes of auricular point acupressure to manage chronic low back pain: A 29-day randomized control study. *Pain Medicine.* 2015;16(10):1857-1869.
15. Yeh CH, Morone NE, Chien LC, et al. Auricular point acupressure to manage chronic low back pain in older adults: A randomized controlled pilot study. *Evidence-Based Complementary and Alternative Medicine.* 2014:Article ID 375173.
16. Yeh CH, Chien LC, Balaban D, et al. A randomized clinical trial of auricular point acupressure for chronic low back pain: A feasibility study. *Evidence-Based Complement Alternative Medicine.* 2013;Article ID 196974:9 pages.
17. Yeh CH, Chien LC, Chiang YC, Huang LC. Auricular point acupressure for chronic low back pain: A feasibility study for 1-week treatment. *Evidence-Based Complement Alternative Medicine.* 2012;2012:Article ID 383257.
18. Yeh CH, Chien LC, Chiang YC, Suen L, Ren D. Analgesic effect of auricular point acupressure as an adjunct treatment for cancer patients with pain. *Pain Management Nursing.* 2015;16(3):285-293.

19. Yeh CH, Chien LC, Lin WC, Bovbjerg DH, van Londen G. Pilot randomized controlled trial of auricular point acupressure to manage symptom clusters of pain, fatigue, and disturbed sleep in breast cancer patients. *Cancer Nursing.* 2016;39(5):402-410.
20. Yeh CH. L, N., Campbell, C., Sair. H., Zhang, F., Mensaha, S., Garry, C., Zeng, J., Chen, C., Pinedoa, M., Khoshnoodi, M., Smith, T.J., Saligan, L.N. Preliminary effectiveness of auricular point acupressure on chemotherapy-induced neuropathy: Part 2 laboratory-assessed and objective outcomes. *Pain Management Nursing.* 2019;20(6):623-632.
21. Yeh CH, Chiang YC, Hoffman S, et al. Efficacy of auricular therapy for pain management: A systematic review and meta-analysis. *Evidence-Based Complementary and Alternative Medicine.* 2014;Article ID 934670.
22. Asher GN, Jonas DE, Coeytaux RR, et al. Auriculotherapy for pain management: A systematic review and meta-analysis of randomized controlled trials. *Journal of Alternative and Complementary Medicine.* 2011;16(10):1097-1108.
23. Farrar JT, Young Jr JP, LaMoreaux L, Werth JL, Poole RM. Clinical importance of changes in chronic pain intensity measured on an 11-point numerical pain rating scale. *Pain.* 2001;94(2):149-158.
24. Dworkin RH, Turk DC, Wyrwich KW, et al. Interpreting the clinical importance of treatment outcomes in chronic pain clinical trials: IMMPACT recommendations. *The Journal of Pain.* 2008;9(2):105-121.
25. Sisignano M, Baron R, Scholich K, Geisslinger G. Mechanism-based treatment for chemotherapy-induced peripheral neuropathic pain. *Nature reviews Neurology.* 2014;10(12):694-707.
26. Smith EM, Bridges CM, Kanzawa G, et al. Cancer treatment-related neuropathic pain syndromes--epidemiology and treatment: an update. *Current pain and headache reports.* 2014;18(11):459.
27. Pike CT, Birnbaum HG, Muehlenbein CE, Pohl GM, Natale RB. Healthcare costs and workloss burden of patients with chemotherapy-associated peripheral neuropathy in breast, ovarian, head and neck, and nonsmall cell lung cancer. *Chemotherapy research and practice.* 2012;2012:913848.
28. Winters-Stone KM, Horak F, Jacobs PG, et al. Falls, Functioning, and Disability Among Women With Persistent Symptoms of Chemotherapy-Induced Peripheral Neuropathy. *Journal of clinical oncology : official journal of the American Society of Clinical Oncology.* 2017;35(23):2604-2612.
29. Flatters SJ, Bennett GJ. Studies of peripheral sensory nerves in paclitaxel-induced painful peripheral neuropathy: evidence for mitochondrial dysfunction. *Pain.* 2006;122(3):245-257.
30. Costigan M, Scholz J, Woolf CJ. Neuropathic pain: a maladaptive response of the nervous system to damage. *Annual review of neuroscience.* 2009;32:1-32.
31. Janes K, Esposito E, Doyle T, et al. A3 adenosine receptor agonist prevents the development of paclitaxel-induced neuropathic pain by modulating spinal glial-restricted redox-dependent signaling pathways. *Pain.* 2014;155(12):2560-2567.
32. Peters CM, Jimenez-Andrade JM, Kuskowski MA, Ghilardi JR, Mantyh PW. An evolving cellular pathology occurs in dorsal root ganglia, peripheral nerve and spinal cord following intravenous administration of paclitaxel in the rat. *Brain research.* 2007;1168:46-59.
33. Ji XT, Qian NS, Zhang T, et al. Spinal astrocytic activation contributes to mechanical allodynia in a rat chemotherapy-induced neuropathic pain model. *PloS one.* 2013;8(4):e60733.
34. Tsavaris N, Kopterides P, Kosmas C, et al. Gabapentin monotherapy for the treatment of chemotherapy-induced neuropathic pain: a pilot study. *Pain medicine (Malden, Mass).* 2008;9(8):1209-1216.
35. Rao RD, Michalak JC, Sloan JA, et al. Efficacy of gabapentin in the management of chemotherapy-induced peripheral neuropathy: a phase 3 randomized, double-blind, placebo-controlled, crossover trial (N00C3). *Cancer.* 2007;110(9):2110-2118.
36. Magnowska M, Izycka N, Kapola-Czyz J, et al. Effectiveness of gabapentin pharmacotherapy in chemotherapy-induced peripheral neuropathy. *Ginekologia polska.* 2018;89(4):200-204.
37. Bellingham GA, Peng PW. Duloxetine: a review of its pharmacology and use in chronic pain management. *Regional anesthesia and pain medicine.* 2010;35(3):294-303.

38. Irwin ML, Cartmel B, Gross CP, et al. Randomized Exercise Trial of Aromatase Inhibitor-Induced Arthralgia in Breast Cancer Survivors. *Journal of clinical oncology : official journal of the American Society of Clinical Oncology.* 2014;33(10):1104-1111.
39. Lu W, Giobbie-Hurder A, Freedman RA, et al. Acupuncture for Chemotherapy-Induced Peripheral Neuropathy in Breast Cancer Survivors: A Randomized Controlled Pilot Trial. *The oncologist.* 2019.
40. Li K, Giustini D, Seely D. A systematic review of acupuncture for chemotherapy-induced peripheral neuropathy. *Current oncology (Toronto, Ont).* 2019;26(2):e147-e154.
41. Franconi G, Manni L, Schroder S, Marchetti P, Robinson N. A systematic review of experimental and clinical acupuncture in chemotherapy-induced peripheral neuropathy. *Evidence-based complementary and alternative medicine : eCAM.* 2013;2013:516916.
42. Smith TJ, Razzak AR, Blackford AL, et al. A Pilot Randomized Sham-Controlled Trial of MC5-A Scrambler Therapy in the Treatment of Chronic Chemotherapy-Induced Peripheral Neuropathy (CIPN). *Journal of palliative care.* 2019:825859719827589.
43. Pachman DR, Weisbrod BL, Seisler DK, et al. Pilot evaluation of Scrambler therapy for the treatment of chemotherapy-induced peripheral neuropathy. *Supportive care in cancer : official journal of the Multinational Association of Supportive Care in Cancer.* 2015;23(4):943-951.
44. Gewandter JS, Chaudari J, Ibegbu C, et al. Wireless transcutaneous electrical nerve stimulation device for chemotherapy-induced peripheral neuropathy: an open-label feasibility study. *Supportive care in cancer : official journal of the Multinational Association of Supportive Care in Cancer.* 2019;27(5):1765-1774.
45. Galantino ML, Brooks J, Tiger R, Jang S, Wilson K. Effectiveness of Somatic Yoga and Meditation: A Pilot Study in a Multicultural Cancer Survivor Population with Chemotherapy-Induced Peripheral Neuropathy. *International journal of yoga therapy.* 2019.
46. Kono T, Mamiya N, Chisato N, et al. Efficacy of goshajinkigan for peripheral neurotoxicity of oxaliplatin in patients with advanced or recurrent colorectal cancer. *Evidence-based complementary and alternative medicine : eCAM.* 2011;2011:418481.
47. Li Y, Cui HJ, Huang JC, Wu XQ. Clinical study of Jiawei Huangqi Guizhi Wuwu Decoction in preventing and treating peripheral neuro-sensory toxicity caused by oxaliplatin. *Chinese journal of integrative medicine.* 2006;12(1):19-23.
48. Liu Y, May BH, Zhang AL, et al. Integrative Herbal Medicine for Chemotherapy-Induced Peripheral Neuropathy and Hand-Foot Syndrome in Colorectal Cancer: A Systematic Review and Meta-Analysis. *Integrative cancer therapies.* 2019;18:1534735418817833.
49. Tofthagen C, Gonzalez L, Visovsky C, Akers A. Self-management of oxaliplatin-related peripheral neuropathy in colorectal cancer survivors. *Chemotherapy research and practice.* 2013;2013:547932.
50. Molsberger AF, Schneider T, Gotthardt H, Drabik A. German Randomized Acupuncture Trial for chronic shoulder pain (GRASP) - a pragmatic, controlled, patient-blinded, multi-centre trial in an outpatient care environment. *Pain.* 2010;151(1):146-154.
51. Nahin RL, Barnes PM, Stussman BJ. Insurance Coverage for Complementary Health Approaches Among Adult Users: United States, 2002 and 2012. *NCHS data brief.* 2016(235):1-8.
52. Schapira MM, Mackenzie ER, Lam R, et al. Breast cancer survivors willingness to participate in an acupuncture clinical trial: A qualitative study. *Support Care Cancer.* 2013;[Epub ahead of print].
53. Nogier P. *Handbook to auriculotherapy.* 1st ed. Moulins-les-Metz: Maisonneuve; 1981.
54. Nogier R. How did Paul Nogier establish the map of the ear? *Medical Acupuncture.* 2014;26(2):76-83.
55. Nogier P. *Points Reflexes Auricularis.* Maisonneuve SA: Moulin Les-Metz, France; 1987.
56. Yeh CH, Huang LC. Comprehensive and systematic auricular diagnosis protocol. *Medical Acupuncture* 2013;25(6):423-436.
57. Oleson T. *Auriculotherapy Manual: Chinese and Western Systems of Ear Acupuncture.* 4th ed. Edinburgh: Churchill Livingstone, Elsevier; 2014.

58. Huang LC. *Auricular Medicine: A Complete Manual of Auricular Diagnosis and Treatment.* 1st ed. Orlando Florida: Auricular International Research & Training; 2005.
59. Alimi D, Geissmann A, Gardeur D. Auricular acupuncture stimulation measured on functional magnetic resonance imaging. *Medical Acupuncture.* 2002;13(2):18-21.
60. Romoli M, Allais G, Airola G, et al. Ear acupuncture and fMRI: a pilot study for assessing the specificity of auricular points. *Neurological Sciences.* 2014;35 Suppl 1:189-193.
61. Keller M, Mazuch J, Abraham U, et al. A circadian clock in macrophages controls inflammatory immune responses. *Proceedings of the National Academy of Sciences of the United States of America.* 2009;106(50):21407-21412.
62. Fonken LK, Frank MG, Kitt MM, Barrientos RM, Watkins LR, Maier SF. Microglia inflammatory responses are controlled by an intrinsic circadian clock. *Brain, behavior, and immunity.* 2015;45:171-179.
63. Vickers AJ, Cronin AM, Maschino AC, et al. Acupuncture for chronic pain: Individual patient data meta-analysis. *Archives of internal medicine.* 2012;172(19):1444-1453.
64. Vickers AJ, Vertosick EA, Lewith G, et al. Acupuncture for Chronic Pain: Update of an Individual Patient Data Meta-Analysis. *The journal of pain : official journal of the American Pain Society.* 2018;19(5):455-474.
65. Lundeberg T, Lund I. Acupuncture for preconditioning of expectancy and/or Pavlovian extinction. *Acupuncture in Medicine.* 2008;26(4):234-238.
66. Lundeberg T, Lund I, Naslund J. Acupuncture - Self-appraisal and the reward system. *Acupuncture Medicine.* 2007;25(3):87-99.
67. Furlan AD, Yazdi F, Tsertsvadze A, et al. A systematic review and meta-analysis of efficacy, cost-effectiveness, and safety of selected complementary and alternative medicine for neck and low-back pain. *Evidence-Based Complement Alternative Medicine.* 2012;Article ID 953139:61 pages.
68. Vickers AJ, Cronin AM, Maschino AC, et al. Individual patient data meta-analysis of acupuncture for chronic pain: protocol of the Acupuncture Trialists' Collaboration. *Trials.* 2010;11:90.
69. Hsu C, Sherman KJ, Eaves ER, et al. New perspectives on patient expectations of treatment outcomes: Results from qualitative interviews with patients seeking complementary and alternative medicine treatments for chronic low back pain. *BMC Complement Altern Med.* 2014;14(276).
70. Schafer LM, Hsu C, Eaves ER, et al. Complementary and alternative medicine (CAM) providers' views of chronic low back pain patients' expectations of CAM therapies: a qualitative study. *BMC Complement Altern Med.* 2012;12:234.
71. Schnur JB, Hallquist MN, Bovbjerg DH, Silverstein JH, Stojceska A, Montgomery GH. Predictors of expectancies for post-surgical pain and fatigue in breast cancer surgical patients. *Pers Individ Dif.* 2007;42(3):419-429.
72. Lind BK, Lafferty WE, Tyree PT, Sherman KJ, Deyo RA, Cherkin DC. The role of alternative medical providers for the outpatient treatment of insured patients with back pain. *Spine.* 2005;30(12):1454-1459.
73. Shelton RC, Clarke Hillyer G, Hershman DL, et al. Interpersonal influences and attitudes about adjuvant therapy treatment decisions among non-metastatic breast cancer patients: an examination of differences by age and race/ethnicity in the BQUAL study. *Breast cancer research and treatment.* 2013;137(3):817-828.
74. Shimozuma K, Ohashi Y, Takeuchi A, et al. Feasibility and validity of the Patient Neurotoxicity Questionnaire during taxane chemotherapy in a phase III randomized trial in patients with breast cancer: N-SAS BC 02. *Supportive care in cancer : official journal of the Multinational Association of Supportive Care in Cancer.* 2009;17(12):1483-1491.
75. Craig BM, Reeve BB, Brown PM, et al. US valuation of health outcomes measured using the PROMIS-29. *Value Health.* 2014;17(8):846-853.
76. PROMIS-29 Profile v2. http://www.assessmentcenter.net/documents/InstrumentLibrary.pdf. Accessed July 31, 2015.

77. (CMS) CfMMS. The 2019 Rate Announcement and Call Letter:and selecting "2019 Announcement.". 2018; https://www.cms.gov/Medicare/Health-Plans/MedicareAdvtgSpecRateStats/Announcements-and-Documents.html Accessed May 24, 2018.
78. Campbell CM, Carroll CP, Kiley K, et al. Quantitative sensory testing and pain-evoked cytokine reactivity: comparison of patients with sickle cell disease to healthy matched controls. *Pain.* 2016;157(4):949-956.
79. Yoo SS, Teh EK, Blinder RA, Jolesz FA. Modulation of cerebellar activities by acupuncture stimulation: evidence from fMRI study. *NeuroImage.* 2004;22(2):932-940.
80. Chen X, Spaeth RB, Freeman SG, et al. The modulation effect of longitudinal acupuncture on resting state functional connectivity in knee osteoarthritis patients. *Molecular pain.* 2015;11.
81. Yeh CH, Chien LC, Albers KM, et al. Function of auricular point acupressure in inducing changes in inflammatory cytokines during chronic low back pain: A pilot study. *Medical Acupuncture.* 2014;26(1):31-39.
82. Agresti A. *Categorical Data Analysis.* 2nd ed. New Jersey: John Wiley & Sons Inc.; 2002.
83. Verbeke G, Molenberghs G. *Linear Mixed Models for Longitudinal Data.* Springer Science & Business Media; 2009.
84. Xiang L, Tse SK, Lee AH. Influence diagnostics for generalized linear mixed models: applications to clustered data. *Computational Statistics & Data Analysis.* 2002;40(4):759-774.
85. Shrout PE, Bolger N. Mediation in experimental and nonexperimental studies: new procedures and recommendations. *Psychological methods.* 2002;7(4):422-445.
86. Lin WC, Burke LE, Schlenk EA, Yeh CH. Use of an Ecological Momentary Assessment Application to Assess the Effects of Auricular Point Acupressure for Chronic Low Back Pain. *CIN: Computers, Informatics, Nursing.* 2019;37(5):276-282.
87. Zeger SL, Liang KY, Albert PS. Models for longitudinal data: a generalized estimating equation approach. *Biometrics.* 1988;44(4):1049-1060.
88. Birhanu T, Molenberghs G, Sotto C, Kenward MG. Doubly robust and multiple-imputation-based generalized estimating equations. *Journal of biopharmaceutical statistics.* 2011;21(2):202-225.
